# Characterization of primary human leptomeningeal cells in 2D culture

**DOI:** 10.1016/j.heliyon.2024.e26744

**Published:** 2024-02-20

**Authors:** Mannthalah Abubaker, Aisling Greaney, David Newport, John J.E. Mulvihill

**Affiliations:** a Bernal Institute, University of Limerick, Castletroy, Limerick, Ireland; b School of Engineering, University of Limerick, Castletroy, Limerick, Ireland

**Keywords:** Leptomeningeal cells, Meningeal barriers, Cerebrospinal fluid, Barrier cells, Transendothelial/transepithelial electrical resistivity, Morphology, Arachnoid mater, Pia mater

## Abstract

Maintaining the integrity of brain barriers is critical for a healthy central nervous system. While extensive research has focused on the blood-brain barrier (BBB) of the brain vasculature and blood-cerebrospinal fluid barrier (BCSFB) of the choroid plexus, the barriers formed by the meninges have not received as much attention. These membranes create a barrier between the brain and cerebrospinal fluid (CSF), as well as between CSF and blood. Recent studies have revealed that this barrier has been implicated in the development of neurological and immunological disorders. In order to gain a deeper comprehension of the functioning and significance of the meningeal barriers, sophisticated models of these barriers, need to be created. The aim of this paper is to investigate the characteristics of commercially available primary leptomeningeal cells (LMCs) that form the meningeal barriers, in a cultured environment, including their morphology, proteomics, and barrier properties, and to determine whether passaging of these cells affects their behaviour in comparison to their *in vivo* state. The results indicate that higher passage numbers significantly alter the morphology and protein localisation and expression of the LMCs. Furthermore, the primary cell culture co-stained for S100A6 and E-cadherin suggesting it is a co-culture of both pial and arachnoid cells. Additionally, cultured LMCs showed an increase in vimentin and cytokeratin expression and a lack of junctional proteins localisation on the cell membrane, which could suggest loss of epithelial properties due to culture, preventing barrier formation. This study shows that the LMCs may be a co-culture of pial and arachnoid cells, that the optimal LMC passage range is between passages two and five for experimentation and that the primary human LMCs form a weak barrier when in culture.

## Graphical abstract

**Figure:**
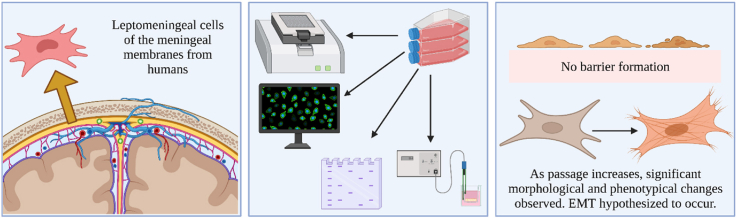

## 1 Introduction

The cells and tissue barriers of the brain are increasingly being recognized for their important role in maintaining the developed central nervous system [[1], [2], [3], [4]]. The central nervous system cells and tissue barriers include [1] the blood-brain barrier (BBB) [2], the blood-cerebrospinal fluid barrier (BCSFB) [3], the arachnoid barrier, and [4] the outer CSF-brain barriers, typically referred to as the pia mater or pia-arachnoid complex when including the subarachnoid space (SAS) [5,6]. However, unlike the BBB and BCSFB, the arachnoid and pia barriers, to be referred to as the meningeal barriers, remain comparatively understudied. The meninges are the membranous tissue, made up of the dura mater, the arachnoid mater and the pia mater, which provide mechanical, immunological, and various other rising roles to the brain parenchyma [[7], [8], [9], [10]]. An increasing body of research is revealing an important role for these meningeal barriers in the development and progression of pathological and neurological disorders, particularly in conjunction with alterations in CSF flow and impact of CSF flow on the accumulation of plaque and toxins at the interfaces of these barriers [[11], [12], [13], [14], [15]]. One prominent example includes the advances in the area of glymphatic system involved in CSF flow and removal of waste through meningeal lymphatic vessels [16]. Despite the fact that CSF is mainly confined to the subarachnoid space and in continuous contact with the meningeal barriers, only a limited amount of research has been carried out on the interaction of the meninges tissue and its resident cells with the CSF, and the role of the cell and tissue barriers of the meninges in the developed human brain.

Like all biological barriers, the meningeal barriers contain a wide variety of cell types but are mainly formed by fibroblast like cells commonly referred to as the leptomeningeal cells (LMCs) or meningothelial cells (MECs) [10]. The ultrastructure and immunocytochemistry of LMCs can vary depending on their location in the central nervous system and the structures they are associated with [17,18]. However, regardless of their location and associated structures, LMCs function as barriers in the same way. The arachnoid barrier is formed primarily through tight junctions, whereas the pia and inner arachnoid layer, adjacent to the SAS, form a more permeable barrier through gap junctions [[19], [20], [21]]. Nonetheless, both types of barriers serve to restrict the passage of harmful substances into the central nervous system However, the understanding of the role and response of LMCs as barrier cells in relation to injury and disease is limited. To gain a better understanding of these cells, more accurate *in vitro* models of LMCs need to be developed, yet none exist that fully characterize this cell type.

Previous research in the use of the LMCs as barrier models has elicited counterintuitive results. Cha et al. [22] attempted to use primary meningeal cells and *ex vivo* cultured mouse meningeal tissue to study the cell barrier properties of the meninges, but found that the LMCs in culture showed a loss of cell barrier properties. Neutzner et al. [23] utilized engineering constructs with similar microarchitecture comparable to the SAS which showed successful cell-cell interaction, indicating the need for intervention to produce the barrier forming ability of the cells. However, a study by Calingasan et al. [24] showed that culture alters the properties of LMCs possibly indicating that reported leptomeningeal characterization may differ from *in vivo* characterization. The varying results found demonstrate that in-depth characterization of the primary human LMCs is needed for more accurate experimental applications. Particularly as the majority of the studies into the role and function of the LMCs focuses on the use of animal derived LMCs or cells derived from meningioma tumours [[25], [26], [27], [28], [29], [30], [31]].

The objective of this study is to gain a better understanding of the properties of the commercially available primary human LMCs and investigate the effects altered culture conditions have on the growth, morphological, protein expression and barrier properties of the cell. The proteins of interest examined for this study where vimentin, cytokeratin, desmoplakin I + II, S100A6, CRABP2, e-cadherin, connexin-43and occludin. These proteins have been shown to be expressed in *in vivo* models of the meningeal brain barriers. The aim is to identify the cell type as well as the point in culture at which the variability in the cells' responses starts to occur to inform the ideal conditions to study the LMCs in more complex experiments. Previous studies have shown that cultured primary cells undergo changes in their cellular characteristics, including alterations in morphology and protein expression, due to disruptive processes such as passaging [32,33]. Therefore, careful monitoring and control of the effect of passaging on cell behaviour is necessary to ensure reliable and consistent results.

## 2 Results

### 2.1 Characterisation of leptomeningeal cell type

**Fig. 1:**
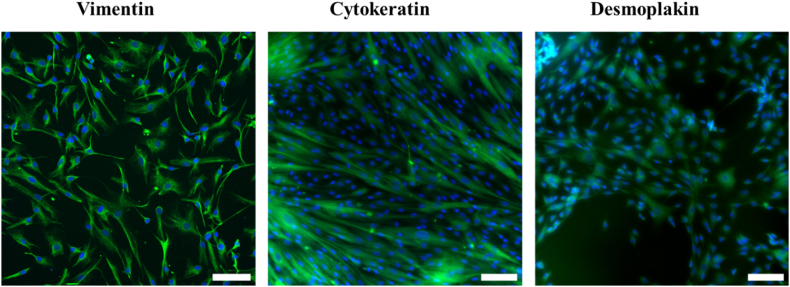
Characterisation of the primary human leptomeningeal cells. Leptomeningeal cells stained for vimentin, cytokeratin and desmoplakin (green) and DAPI (blue). (For interpretation of the references to colour in this figure legend, the reader is referred to the Web version of this article.)

The LMCs were subjected to immunostaining for vimentin, cytokeratin, desmoplakin I + II, S100A6, and CRABP2. The primary goal was to initially determine the cells' origin from the meninges and subsequently determine whether they were arachnoid or pial meningeal cells. Co-staining of S100A6 and CRABP2 was performed in conjunction with e-cadherin to distinguish cells displaying both e-cadherin and CRABP2 positivity as arachnoidal LMCs, while cells demonstrating S100A6 positivity were identified as pial LMCs. Fig. 1 displays immunostained cells for vimentin, cytokeratin, and desmoplakin I + II, all of which exhibited positive staining, confirming their derivation from the meninges.

**Fig. 2:**
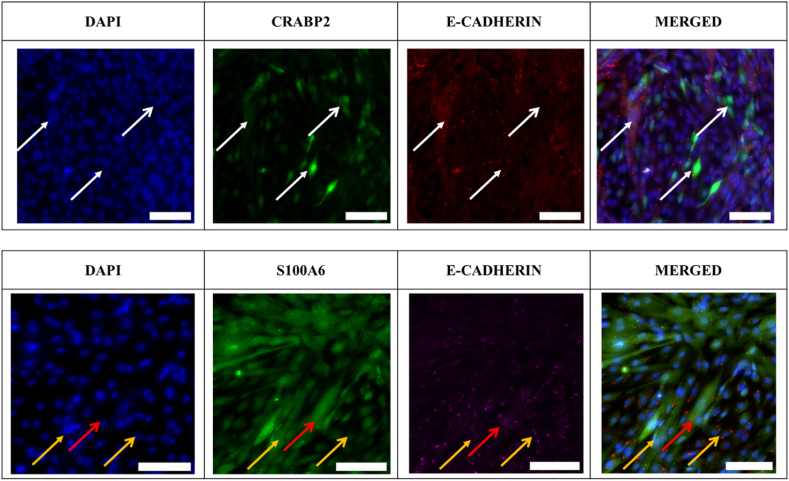
Leptomeningeal cells were stained with CRABP2 and S100A6 to identify meningeal cell type within the culture. CRABP2 and S100A6 (Green) were co-stained with E-cadherin (Red) and DAPI (blue) to identify if cells that stained positive for CRABP2 and E-cadherin (Arachnoidal) and cells that only stain for S100A6 (pial) are found in the culture. The open white arrow head shows CRABP2 only positive cells. The closed white arrow head indicates CRAPB2/E-CAD + cells. The closed yellow arrow head indicates S100A6 only cells. The open yellow arrow head indicates E-cad + cells. The open red arrow head indicates S100A6/E-cad + cells. Scale bar = 100 μm. (For interpretation of the references to colour in this figure legend, the reader is referred to the Web version of this article.)

Fig. 2 presents the results for the cells subjected to the co-staining with S100A6 and e-cadherin, as well as CRABP2 and e-cadherin. The primary objective was to identify any co-localisation between cells exhibiting positive staining for e-cadherin and CRABP2 and/or noting the absence of co-localisation between cells staining for e-cadherin and S100A6. The results indicate positive staining for both CRABP2 and S100A6. Furthermore, they demonstrate that cells positive for both S100A6 and CRABP2 also exhibit positive staining for E-cadherin. However, the staining patterns reveal nuances, emphasizing that not all cells showing positivity for S100A6 and CRABP2 also display positive staining for E-cadherin, as illustrated in the merged figure. A subset of cells exhibits dual positivity for E-cadherin and CRABP2, while another subset exclusively shows CRABP2 positivity. Similarly, there are cells positive for both E-cadherin and S100A6, and cells that solely express S100A6. This indicates the potential presence of a mixed culture comprising both pial and arachnoidal LMCs or a subset of leptomeningeal cells that stain for both.

### 2.2 Leptomeningeal cell growth

**Fig. 3:**
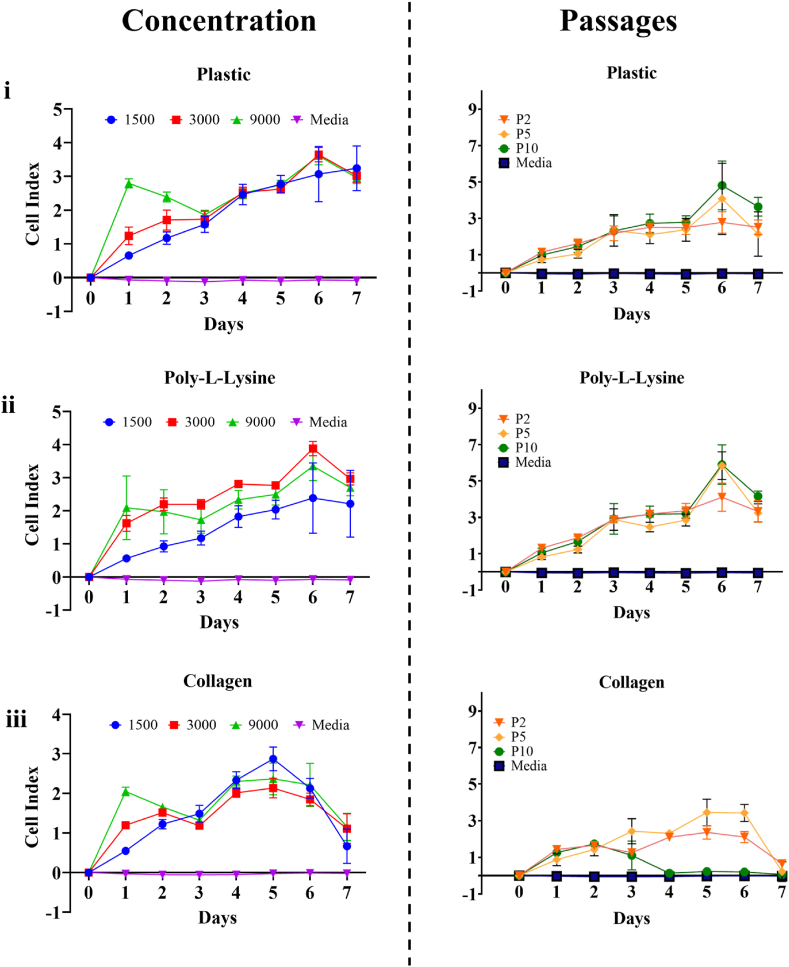
Leptomeningeal cells growth curves at different concentrations and passages, on either non-coated wells (i), or wells coated in poly-l-lysine (ii) or collagen (iii). Leptomeningeal cells were seeded at different concentrations (Left) or passages (Right). Each combination (concentration/passage and substrate) were completed in 3 experimental replicates and 6 technical replicates. Each point represents the mean of the 6 replicates for each day. Cell media was changed on day 3 and day 6. **(Colour)**. (For interpretation of the references to colour in this figure legend, the reader is referred to the Web version of this article.)

Real-time cell analysis was used to measure the LMCs growth at different parameters. Experiments were completed in triplicates. LMC growth at different cell concentrations were first compared at passage two, followed by LMC growth at incremental cell passages. Both cell concentration and cell passages were tested on non-coated wells and wells coated in PLL and collagen to gauge the effect substrates may also have on cell growth (Fig. 3i-iii). LMCs showed rapid attachment and growth over the seven-day period at all concentrations and passages, moving from a cell index (CI) of below one to four or five.

Results of this study show that LMCs seeded at different concentrations do reach different B–CI, although not statistically significant in difference. Specifically, when the LMCs were seeded on PLL, the LMCs at the different cell concentrations had notable variations in B–CI relative to each other (Fig. 3ii). LMCs seeded on non-coated wells and wells coated with collagen, with the exception of a temporary increase observed in the 9x10^4^ cell/mL concentration before day 3, were relatively the same (Fig. 3iii). Comparing the B–CI individually indicated optimal LMC growth occurred when the cells were seeded at a concentration of 3x10^4^ cell/mL on PLL. LMCs grown at this combination showed the highest B–CI relative to the LMCs grown at cell concentrations of 1.5x10^4^ cell/mL and 9x10^4^ cell/mL on non-coated wells or wells coated in collagen (Table 1).

**Table 1:** B–CI values of the LMCs for the different concentrations and passages measured. The values presented are the maximum B–CI shown for each concentration or passage over the 7 days growth, the mean B–CI over the 7 days growth ±S.D, and the mean B–CI at full confluency around day 6 ± S.D.

Concentration									
	Plastic			PLL			Collagen		
	1500	3000	9000	1500	3000	9000	1500	3000	9000
**Maximum B–CI**	3.240	3.644	3.606	2.383	3.877	3.348	2.871	2.131	2.362
**Mean B–CI**	1.944 ± 1.197	2.137 ± 1.141	2.434 ± 1.075	1.346 ± 0.857	2.379 ± 1.145	2.159 ± 0.977	1.437 ± 0.987	1.405 ± 0.682	1.662 ± 0.799
**Day 6 Mean B–CI**	3.135 ± 0.817	3.711 ± 0.214	3.673 ± 0.259	2.450 ± 1.062	3.944 ± 0.217	3.414 ± 0.440	2.139 ± 0.247	1.858 ± 0.159	2.225 ± 0.544
**Passages**									
	**Plastic**			**PLL**			**Collagen**		
	P2	P5	P10	P2	P5	P10	P2	P5	P10
**Maximum B–CI**	2.78	4.076	4.811	4.11	5.843	5.904	2.36	3.448	1.733
**Mean B–CI**	1.950 ± 0.941	1.900 ± 1.249	2.384 ± 1.524	2.557 ± 1.346	2.468 ± 1.797	2.809 ± 1.85	1.476 ± 0.802	1.798 ± 1.349	0.623 ± 0.668
**Day 6 Mean B–CI**	2.819 ± 0.589	4.115 ± 1.954	4.850 ± 1.338	4.149 ± 0.767	5.882 ± 0.764	5.882 ± 1.082	2.12 ± 0.304	3.437 ± 0.725	0.213 ± 0.183

Furthermore, passage wise, the B–CI shown by the larger passages tended to be larger, although when compared showed no statistical significance. LMCs seeded on non-coated wells and PLL yielded similar growth trends for passage two, five and ten (Fig. 3ii). In contrast, LMCs seeded on collagen generated low B–CI with cells at passage 10 showing a decline in viability at day 3 (Fig. 3iii). This was observed on all substrates. The inclusion of the substrates appeared to have the most effect on cell growth and attachment rather than concentration and passage.

### 2.3 Leptomeningeal cell morphology

**Fig. 4:**
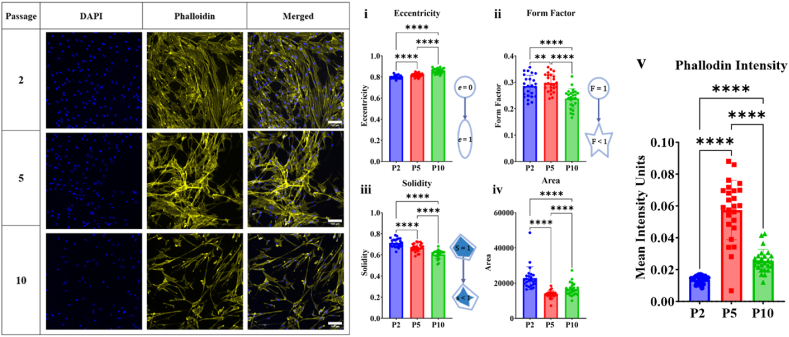
Leptomeningeal cell morphology is impacted by cell passage. Leptomeningeal cells were seeded at a concentration of 3x10^4^ cell/mL and left to grow up to full confluency. Leptomeningeal cells of passage 2, passage 5 and passage 10 were fixed and stained with DAPI (Blue) and Phalloidin (Yellow) and imaged at 20x magnification. A cell imaging software was used to analyse the cells morphology by examining the leptomeningeal cell eccentricity (i), form factor (ii), solidity (iii) area (iv), and phalloidin intensity (v). 25 images were analysed for passage 2, passage 5 and passage 10, each containing an average of around 1000 cells/image. Leptomeningeal cells show significant statistical difference on all morphological parameters. *p < 0.5, **p < 0.01, ***p < 0.001 and ****p < 0.0001. Scale Bar = 100 μm. **(Colour)**. (For interpretation of the references to colour in this figure legend, the reader is referred to the Web version of this article.)

The morphology of LMCs was evaluated by analysing changes in eccentricity, form factor, solidity, and area. A customized pipeline was developed to assess cell morphology based on the chosen parameters, and the results indicated significant differences in cell morphology among cell passages (Fig. 4). Eccentricity is the ratio between the foci of the ellipse and its major axis length, and a value of 1 signifies a more ovular shape with foci further from the centre of the ellipse. LMCs showed a mean normalized ratio of over 0.8 at all passages [P2: 0.799 + 0.015; P6: 0.819 + 0.018; P10: 0.857 + 0.022] suggesting that the cells were elongated and thin rather than circular (Fig. 4i). Form factor, which indicates the shape of an object based on the ratio of its area to perimeter, showed a mean normalized ratio of below 0.4 for all passages of LMCs [P2: 0.286 + 0.041; P6: 0.298 + 0.033; P10: 0.239 + 0.036], implying a fine cellular shape (Fig. 4ii). Solidity, also known as convexity, is the proportion of pixels in the convex hull that are part of the object and can be used to distinguish cells with protrusions or irregular shape from round cells. As solidity approaches 1, the object is more solid and circular, while as it approaches 0, the object is less circular with more protrusions. The LMCs show protrusions at all passages but appears to increase significantly at P10 [P2: 0.713 + 0.039; P6: 0.667 + 0.037; P10: 0.603 + 0.039]. LMCs shows a trend of decreasing in area as the cell passage increases (Fig. 4iii). Thus overall, it appears the cells becomes thinner, longer and showing more protrusions as it increases in cell passage.

Additionally, it is worth noting that morphological differences in the cells become visible microscopically as early as passage five, although not as pronounced as those observed between passage two and ten. The LMCs demonstrate a transition from a highly interconnected and confluent state to a sparsely populated configuration with greater distance between individual cells. This is also confirmed by the decrease in actin intensity at passage 10, suggesting reduced actin co-localisation post passage 5 (Fig. 4v).

### 2.4 Protein expression of leptomeningeal cells

**Fig. 5:**
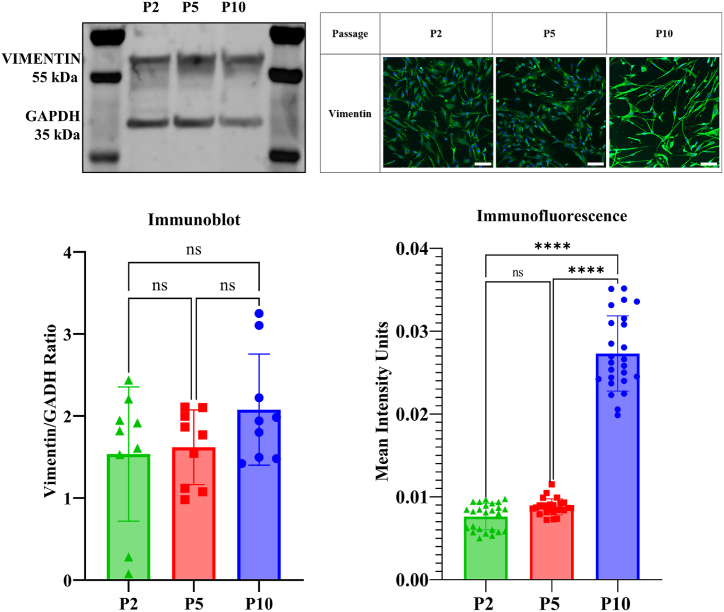
Vimentin was examined for differences depending on cell passage using immunoblot and immunofluorescence. Leptomeningeal cells were seeded at 3x10^4^ cell/mL and either lysed or fixed after reaching full confluence. Immunoblots were completed in triplicates. 25 images were analysed for passage 2, passage 5 and passage 10, each containing an average of 1000 cells/image for the immunofluorescence. Statistical significance was observed in the immunofluorescence intensity between passage 2 and passage 10, and passage 5 and passage 10. No Statistical significance was observed in the immunoblot. *p < 0.05 and ****p < 0.0001. Scale Bar = 100 μm. **(Colour)**. (For interpretation of the references to colour in this figure legend, the reader is referred to the Web version of this article.)

**Fig. 6:**
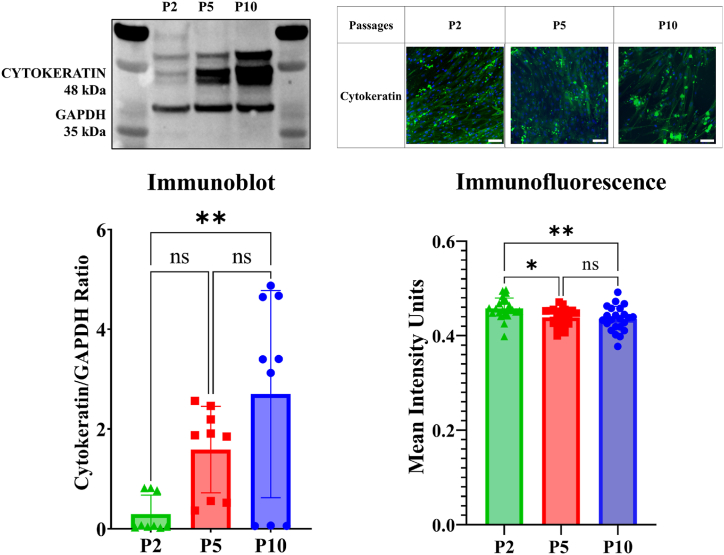
Cytokeratin was examined for differences depending on cell passage using immunoblot and immunofluorescence. Leptomeningeal cells were seeded at 3x10^4^ cell/mL and either lysed or fixed after reaching full confluence. Immunoblots were completed in triplicates. 25 images were analysed for passage 2, passage 5 and passage 10, each containing an average of 1000 cells/image for the immunofluorescence. Statistical significance was observed in the immunofluorescence intensity between passage 2 and passage 5, and passage 2 and passage 10. Statistical significance was observed in the immunoblot between passage 2 and passage 10. *p < 0.05 and ****p < 0.0001. Scale Bar = 100 μm. **(Colour)**. (For interpretation of the references to colour in this figure legend, the reader is referred to the Web version of this article.)

**Fig. 7:**
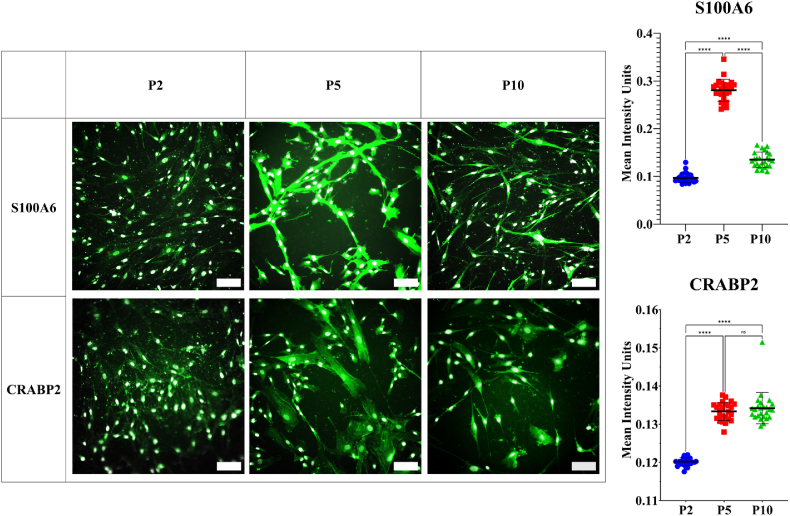
Protein localisation of S100A6 and CRABP2 were examined for leptomeningeal cells using immunofluorescence. Leptomeningeal cells of passage 2, passage 5 and passage 10 were fixed and stained for each protein and imaged at 20x magnification. A cell imaging software was used to analyse the mean intensity for each passage. 25 images were analysed for passage 2, passage 5 and passage 10, each containing an average of 1000 cells/image. Leptomeningeal cells show significant statistical difference between the different passages for S100A6 and CRABP2 indicating the protein localises differently as the cell is passaged. ****p < 0.0001. Scale Bar = 100 μm. **(Colour)**. (For interpretation of the references to colour in this figure legend, the reader is referred to the Web version of this article.)

Protein expression of LMCs was analysed using immunoblots and immunofluorescence at different passages. Vimentin and pan-Cytokeratin were examined using both techniques (as shown in Fig. 5, and Fig. 6), while S100a6 and CRABP2 were analysed only using immunofluorescence (as shown in Fig. 7) due to low molecular size. Immunoblot analysis revealed that vimentin expression increased as passage number increased, although no significant differences in expression between the passages was observed statistically. Immunofluorescence analysis showed that vimentin became more localized in the LMC at higher passages, with statistically significant differences observed between passage two and passage five with passage ten. Results suggest that Vimentin expression increases with passage number and becomes more localized in LMCs at higher passage.

The pan cytokeratin exhibited a molecular weight of 48 kDa, indicative of cytokeratin CK8/18 typically present in epithelial cells. In the context of passage number, the immunoblot analysis revealed a significant increase in protein expression. This increase was particularly pronounced between passage two and passage ten, showing statistical significance. In contrast, the immunofluorescence analysis highlighted a decrease in cytokeratin localisation as passages advanced. Notably, significant differences were observed between passage two and passage five, as well as passage ten. These results propose a similarity to vimentin behaviour, indicating an upsurge in cytokeratin expression with progressive passages. However, unlike vimentin, cytokeratin's localisation diminishes with increasing passage number.

The immunofluorescence analysis of S100A6 and CRABP2 demonstrated a trend of increased localisation with increasing passages. S100A6 expression showed a significant increase at passage five compared to passage two and passage ten, with statistically significant differences observed between all passages. Similarly, CRABP2 expression showed an increase at passage five compared to passage two but remained at a similar localisation level at passage ten. However, statistically significant differences were only observed between passage two and the later passages. These findings suggest that both S100A6 and CRABP2 exhibit a trend of increased localisation with increasing passages.

### 2.5 Barrier integrity of leptomeningeal cells

**Fig. 8:**
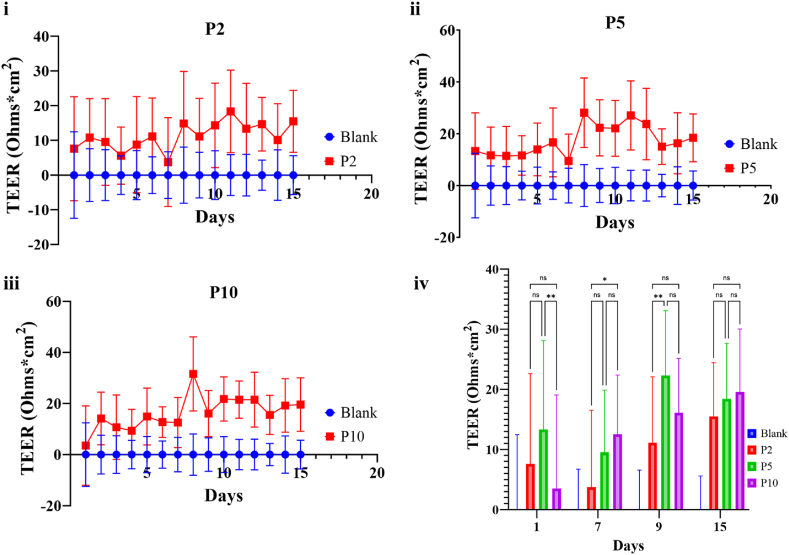
Transendothelial/Transepithelial electrical resistance (TEER) of leptomeningeal cell barrier was measured every day up to 16 days using the EVOM2. (i-iii) Data points represent the mean of triplicate measurements across triplicate wells and triplicate plates (n = 27). Error bars denote standard deviation. (iv) Significant difference is observed on day 1, 7 and 9. *p < 0.5 and **p < 0.01. **(Colour)**. (For interpretation of the references to colour in this figure legend, the reader is referred to the Web version of this article.)

Next, we evaluated the barrier-forming capacity of primary human LMCs in culture and the impact of passaging on their barrier integrity. Transendothelial/transepithelial electrical resistivity (TEER) measurements were taken daily for 16 days (Fig. 8). Results indicated weak barrier formation of these cells when cultured per manufacture recommendations, with no significant differences observed in TEER measurement between the passages. LMCs at passage two showed the lowest maximum TEER values relative to passage five and passage ten (P2: 18.33, P5: 28.08, and P10: 31.53) (Fig. 8iv). TEER measurements peaked at day nine and remained relatively high up to day fifteen (Fig. 8i–iii).

**Fig. 9:**
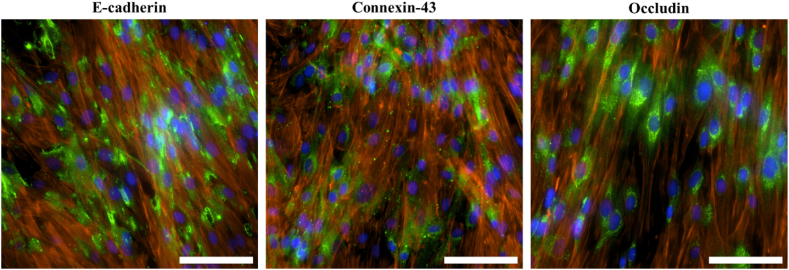
Leptomeningeal cells stained with E-cadherin, Connexin-43 and Occludin (green). Proteins were co-stained with phalloidin (red) and DAPI (blue). Images were taken at 40x magnification. (For interpretation of the references to colour in this figure legend, the reader is referred to the Web version of this article.)

To corroborate the weak barrier formation of the LMCs, we conducted an examination of the protein localisation of three barrier proteins, E-cadherin an adherens junction protein, Connexin-43 a gap junction protein and Occludin a tight junction protein, when the cells were fully confluent on day 7 (Fig. 9).

**Fig. 10:**
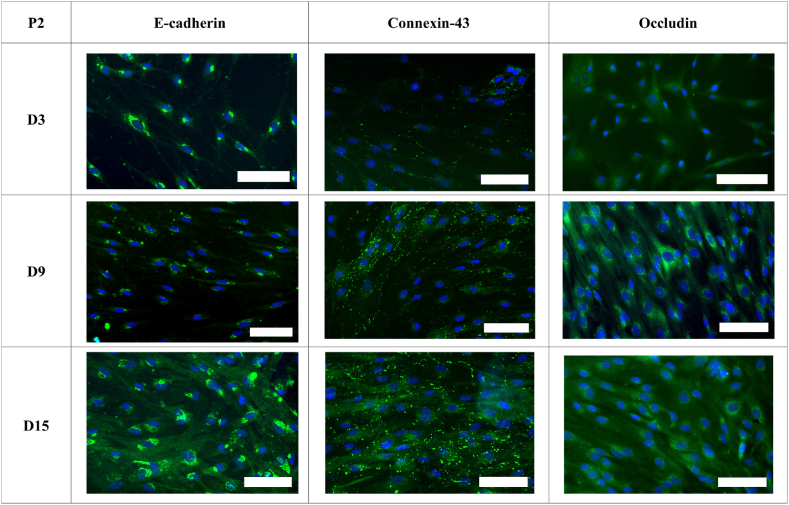
Immunofluorescence staining of junctional proteins E-cadherin (Green stain), Connexin-43 and Occludin on day 3, day 9 and day 15 of leptomeningeal cells in culture to examine if a barrier forms if the cells remain longer in culture. DAPI nuclear stain (blue) was used to identify cells. Images were taken at 20x magnification. **(Colour)**. (For interpretation of the references to colour in this figure legend, the reader is referred to the Web version of this article.)

Staining for e-cadherin, connexin-43, and occludin revealed the presence of these proteins, but their localisation around the cell membrane was not observed, even at full confluency. To delve deeper into this observation, e-cadherin, connexin-43, and occludin were stained on day 3, day 9 and day 15 in LMC P2 to detect potential changes in localisation as the cells grow in culture (Fig. 10).

Immunofluorescence results indicated that while e-cadherin was present within the cells, it did not localise at the cell junctions. This localisation pattern remained consistent across the varying days in culture, showing no significant alterations. Similarly, occludin exhibited a consistent localisation pattern that did not change over time. An extended culture period did not lead to an improvement in the localisation. In contrast, connexin-43 was observed to localise around the cell membrane, in particular at day 15. These findings suggest that the integrity of the LMC barrier primarily relies on adherens and tight junctions.

## 3 Discussion

There has been a growing focus on exploring the involvement and the significance of the LMCs in injury and disease [[34], [35], [36], [37], [38], [39]]. Thus characterising the primary human LMC in 2D culture for experimental purposes is necessary to ensure reliable and reproducible results. Previous work on characterisation of LMCs in culture utilized cells sourced from animals or from a meningioma cell line [26,31,[40], [41], [42]]. As the significance of LMCs in human health gains prominence, the utilization of experimental techniques involving human cells becomes increasingly prevalent.

Our study focused on obtaining primary human LMCs from Sciencell [#1400] and characterising them for future experimental use. This consisted of evaluating the barrier properties of these cells in culture, their morphology, protein expressions and growth rate.

### 3.1 Characterisation of leptomeningeal cell type

*In vivo*, LMCs play a pivotal role in forming both the arachnoid and pial barriers, effectively preventing unregulated passage of substances and pathogens between the central nervous system and surrounding tissues [19,26]. The identification of LMCs has traditionally relied on positive staining for vimentin, cytokeratin, and desmoplakin [17,43,44]. More recently, the expression of S100A6 and CRABP2 has been reported as additional markers that can distinguish between arachnoid and pial cells [18]. Vimentin, a key structural component of the cytoskeleton, and cytokeratin, present in the intracytoplasmic cytoskeleton of epithelial tissue, are widely utilized markers for LMC identification [45,46]. Desmoplakin, the primary component of desmosomes found in meninges, plays a critical role in cell-cell adhesion across various cell types and is particularly important in tissues subject to mechanical stress [[46], [47], [48]].

The commercially obtained primary human LMCs utilized in this study displayed positive staining for vimentin, cytokeratin, and desmoplakin, albeit without strong barrier staining. The role of vimentin in LMCs remains incompletely characterized, but it can be hypothesized that its expression is linked to mechanical strain and protective functions [[49], [50], [51]]. The meninges primarily serve as a protective tissue, absorbing substantial forces to safeguard the brain and spinal cord, providing a plausible explanation for the presence of vimentin in these cells. In the subsequent section of this study, the cytokeratin identified was specifically cytokeratin 8/18. CK 8/18 represents a pair of intermediate filament proteins primarily found in simple epithelial cells. These proteins are typically associated with cells forming a single layer of closely packed cells, engaged in processes such as secretion, absorption, and protection [52]. The presence of cytokeratin in LMCs has been a subject of debate, with some cells expressing it while others do not. This variability may be attributed to the specific location of LMC derivation and their respective roles *in vivo* [53]. Desmoplakin, on the other hand, is primarily associated with arachnoid cells and arachnoid villi cells [17,54]. While it is also associated with pial LMCs, its presence is comparatively limited, likely due to the pia's well-known weaker barrier properties compared to the arachnoid. Intriguingly, LMCs derived from the optic nerve were found to exhibit no immunopositivity for desmoplakin [55].

Moreover, S100A6 and CRABP2 in LMCs are currently employed to differentiate these cells although their role in the LMCs is unknown [18,56,57]. In our study, the LMCs exhibited positive staining for both proteins, suggesting the potential presence of a mixed culture comprising both pial and arachnoidal cells or intermediate cell type. Analysis of the developing meninges and mouse meningeal models showed that the heterogeneity of the meninges is complex. For example, the pial meninges in the cortex can be both negative and positive for S100A6 while the arachnoid meninges could be positive for S100A6(18,56). Similarly, the arachnoid meninges are comprised of the arachnoid barrier cells and inner arachnoid layer, each distinct in cell type with different gene and protein expression. It has also been shown that certain genes and proteins change from the developmental meninges to the adult meninges further explaining why we see a positive stain for S100A6 in e-cadherin positive cells, as in human LMCs there may be a need for these cells to express S100A6 that is unknown. While further analysis is required to make a definitive distinction, this information holds significance for experimental purposes. If this is indeed a co-culture of primary cells, then cell sorting becomes necessary to more effectively isolate the distinct cell types.

Consequently, the choice of LMCs for experimentation should align with the research objectives. If the aim is to study the barrier functions of LMCs, arachnoid cells may be more suitable [20]}. Conversely, if the research focuses on understanding the role of LMCs in interactions with the brain, pial cells may be the preferable choice. The decision should be guided by the specific goals and context of the experiment.

### 3.2 Leptomeningeal cell growth

Previous work on growth characterization of the LMCs have found that these cells (animal and human derived) exhibit slow growth, with cells reaching confluency at around seven days to two weeks [45,46,[58], [59], [60]]. This gradual growth trend was confirmed in primary human LMCs within this study. Additionally, it has been noted that the LMCs reach confluency at lower cell densities compared to fibroblasts and astrocytes suggesting the LMCs have a unique growth pattern and behaviour compared to other cell types [45,46]. This was further confirmed by comparing the LMC max B–CI to that of DITNC1 astrocytes (Supplementary Figure S1-S9) where astrocytes were observed to reach confluency on day 3 with a max B–CI of 7–8 in comparison to the LMCs that reach confluency on day 6 with a max B–CI of 4–5. Interpretation of the B–CI values presented were completed according to Kho et al. [61] in which it was reported that a cell index value of 1–4 shows weak cell attachment, 5–10 moderate cell attachment, and 12–15 strong cell attachment. LMC adhesion ranked low to moderate at full confluency, with mean B–CI consistently below six across all tested combinations. LMC growth analysis revealed consistent proliferation irrespective of cell concentration and passages. Cells reached a uniform proliferation peak on day six before entering a stationary phase on day seven. Slight decline observed by the system around and after day 7 (Supplementary Figure S1) is indication of changes occurring to the cell related to reduction in focal adhesion [61]. This could be an indication of when cellular compromise is beginning, which is marked by a gradual or progressive decline in the CI. Such a trend is important when conducting long term experimental procedures. However, for the LMCs, there is no decline but a plateau after each media change where the cells stay around the same CI. This suggests robust contact inhibition in LMCs, unique as not all cell types exhibit this trait. The unaffected proliferation by cell concentration or passages indicates a stable growth behaviour.

Additionally, substrate type influenced LMC proliferation. Given their natural fibrous, collagen-rich *in vivo* environment [51], collagen substrate was expected to promote proliferation. Surprisingly, PLL-coated wells exhibited the highest mean B–CI, implying superior LMC adhesion and proliferation compared to collagen and non-coated wells. Collagen showed the lowest B–CI, suggesting less efficient growth. Several factors might account for the differential response of LMCs to collagen as a substrate in the described experiment. One potential factor is the type or concentration of collagen employed, potentially influencing the outcomes. Notably, both collagen and PLL have demonstrated strong adhesive properties, fostering cell proliferation [[62], [63], [64]]. However, the unexpectedly low B–CI values observed with collagen imply the involvement of other influencing factors. Future research could investigate the effect of different substrate concentrations on LMC growth and explore ways to create a more accurate model of the *in vivo* environment in which LMCs exist. This may involve developing new methods for replicating the fibrous and collagenous matrix present *in vivo* or using other techniques to better mimic the physiological conditions that LMCs encounter in the body.

### 3.3 Leptomeningeal cell morphology

Previous literature has described LMCs as flat polygonal cells that are bipolar and large at sub-confluence, and homogonous with cobblestone-like appearance at full confluency [25,45,46,[58], [59], [60]]. The results of this study through immunofluorescence and quantification of the images, confirms the homogeneity of the LMCs at full confluency but did not observe their characteristic cobblestone appearance. Instead, these LMCs displayed a smooth muscle-like morphology with a long and slender shape. This deviation from prior observations may be attributed to differences in the origin of the LMCs used in previous literature, as many of them were derived from animal LMCs or LMCs obtained from meningioma tumours. In addition, the findings from this study validate notable alterations in morphology as LMCs undergo successive passaging. Interestingly, the LMCs become thinner, longer and showing more protrusions as it increases in passage. The LMCs also appear to become sparsely populated at passage 10. Previous reports into the effect of passaging on cells has shown that cultured cells after a certain passage show reduced proliferative capacity and struggle to reach confluency after 1 week under standard culture conditions [65]. This is observed by the LMCs in Fig. 2. This can also be explained by the probability that due to the change in morphology of LMCs at the higher passage, that they appear less dense. Furthermore, F-actin restructure with passage was also observed by measuring phalloidin intensity in the cells. This is a significant observation since cellular responses can be influenced by changes in morphology [66]. The effect of passaging on cell morphology and cytoskeleton restructure has been previously reported in other primary cell cultures such as stromal cells, primate endothelial cells and arterial endothelial cells all showing changes in protein expression profiles, differences in cell spreading, shape and migrations [65,[67], [68], [69]]. These changes in morphology due to passaging can be attributed to a variety of factors. They may be a result of disruption of cell-cell contact and the loss of cell polarity, or due to increasing cell stress associated with passaging leading to changes in shape and structure. It has also been reported that cells after a certain passage when showing signs of stress and exhibiting functional senescence by not reaching confluency no matter how long left in culture as can be seen for the LMCs in Fig. 2 [65]. Visually, the LMCs show significant changes when observed microscopically at the different passages, furthermore confirming the potential injury response of these cells due to culture conditions. The cells become less confluent and showing less cell-cell contact as they near passage ten.

### 3.4 Protein expression of leptomeningeal cells

In this study, all proteins tested were sensitive to passaging. Both vimentin and cytokeratin exhibited changes in expression and localisation with passage number, indicating their sensitivity to passaging. As both vimentin and cytokeratin are intermediate filaments which connect the cell membrane proteins to the cell nucleus, changes in their expression due to passaging may mean changes in the interaction between membrane proteins and the nucleus [52]. In this study, an upregulation of both vimentin and cytokeratin is observed. An upregulation of intermediate filaments is typically associated with a wounded cell state, as the cells work to heal [70]. This can be due to a breakdown of the epithelial barrier, increased cell motility and invasiveness, and resistance to apoptosis [71]. Due to the fibroblast nature of the LMCs, they already express vimentin naturally, but the potential upregulation at the higher passages may be due to the aforementioned reasons, still indicative of a changed cell state. Similarly, it has been reported that an increase in cytokeratin expression in cell culture has been attributed to epithelial cells entering an atypical, active state [72]. However, due to the mixed nature of LMCs being epithelial fibroblast-like cells, it can be hypothesized that as the cells increase in passage, more cytokeratin is required to promote cell-cell adhesion and vimentin for resistance to migration-related stress [73].

Previous literature reports state a continuous vimentin expression across all passages, but a loss of cytokeratin. However, the localisation pattern undergoes changes with increasing passages, transitioning from perinuclear staining in the early passages to a more diffusely cytoplasmic distribution in the later passages, as seen by our study [45,46]. Furthermore, S100A6 showed an increased mean intensity at P5 and an overall significant difference between all passages. This is interesting as a similar increased mean intensity is observed for F-actin filaments of the cells at P5, possibly confirming the role of S1006 as a regulator of actin organisation in the LMCs [74]. In pulmonary fibroblast, S100A6 was shown to not only promote cell proliferation, but was also involved in many other major cells process and had a role in determining cell morphology and cytoskeletal organization [75]. CRABP2, on the other hand, showed no significant difference in mean intensity between passages. These changes in the localisation of S100A6 and CRABP2 in the LMCs could be an indication of metabolic changes occurring within the cells as it increases in passage. Similarly, CRABP2 have also shown to have a role in elastin expression, wound re-epithelialization and has been shown to be mandatory for cell survival [[76], [77], [78]]. Overall, a more comprehensive understanding of the changes in protein expression observed in LMCs at different passages is required.

### 3.5 Barrier integrity of leptomeningeal cells

Our findings suggest that even after being cultured to full confluency for a 15-day duration, primary human LMCs did not demonstrate barrier properties or an intact cellular layer. Instead, the TEER values indicated a compromised barrier or the presence of weak junctions. Previous studies into the barrier integrity of the cells used immortalized arachnoid cells line from rats measured max TEER values of 168 ± 3.9 Ohms/cm^2^ much higher than the values obtained for the primary LMCs [79]. LMCs derived from tumour biopsies showed TEER values in the range of this study, not generating a TEER higher than 37 ± 17 Ohms/cm^2^ [80]. To the authors knowledge, no further studies have been conducting on improving the TEER values of the LMCs. Similarly, an *in vivo* TEER quantification of the meningeal barriers for comparison is also yet to be reported. This indicates a gap in our understanding of the meningeal barriers and a limitation to creating a mimic model of the barrier for further experimentation.

To delve deeper into the underlying reasons for the low TEER values of the LMCs, we conducted a study examining the localisation of three key junctional proteins: e-cadherin, connexin-43 and occludin, over a period of fifteen days. E-cadherin, a member of the cadherin family, is an essential cell adhesion molecule that plays a role in forming adherens junctions and maintaining cell-cell contact and stability. Connexin-43, a gap junction protein, has been found in the meninges and their projections into the brain. Occludin is a tight junction protein and has been found to be important in tight junction stability and barrier function [81,82].

E-cadherin and occludin, although present in the cell, appeared to localise mainly around the cell nucleus. Absence of e-cadherin has been shown to lead to improper localisation of key tight junctional proteins such as occludin, resulting in permeable barriers [83]. Similarly, it has also been shown that e-cadherin mediated cell-cell adhesion is not essential for the assembly of gap junctions such as connexin-43 [84]. Connexin-43 in the LMCs appeared to localise at the cell membrane, forming cell to cell junctions. Connexin-43 was also found in larger quantities in the LMCs on day 15 of culture in comparison to the earlier days in culture. Previous studies have also reported the loss of junctional proteins in cultured primary LMCs [22,80]. Similar results were also seen in cultured arachnoid granulation cells in which connexin-43 showed punctate like staining [53]. Therefore, the localisation of these junctional proteins helps explain why primary human LMCs in culture form a weak barrier.

This knowledge is crucial because it underscores the fact that primary human LMCs in culture do not accurately reflect the dynamics of the *in vivo* meningeal barrier. Several factors may explain why LMCs exhibit this behaviour in culture. Firstly, the culture environment does not faithfully replicate the *in vivo* conditions of these cells. For instance, meningeal tissue possesses a Young's modulus of 9.43 MPa [85], whereas the Youngs modulus of most cell culture plastics is around 1 GPa [86]. Thus, growing the cells on a substrate more closely resembling the properties of meninges may facilitate the initiation of barrier functions, as observed in other cell types [87].

Moreover, the meninges experience shear stress due to the movement of CSF within the SAS [[88], [89], [90]]. Similar to endothelial cells that necessitate shear stress to establish impermeable barriers, it is conceivable that LMCs might also require this mechanical stimulation [91].

### 3.6 Limitations and future work

While this study has shed light on the behaviour of LMCs in culture, there are still some limitations that need to be addressed. Firstly, this study relied on a single vial of commercially obtained primary human LMCs for characterisation in experimental applications. To enhance the robustness of the findings, it is advisable to conduct additional replicates using two more vials. Furthermore, as the LMCs appear to be a co-culture, we recommend further testing to determine meningeal subtype using more specific makers to distinguish between the types of cells. Thus, cell sorting may become necessary to more efficiently isolate the cell types.

Secondly, the LMCs were grown on PLL and future work should include a substrate memetic of the ECM of the meninges. It is possible that the cells may react differently to varying substrate concentrations and types. Therefore, further experimentation is necessary to establish a more comprehensive understanding of the factors influencing the behaviour of the LMCs.

While the study has used pan-Cytokeratin as a marker for the epithelial-like phenotype of the cells, a more specific analysis of cytokeratin is required to understand the specific function of each subtype. This will provide further insight into the role of LMCs in the formation and maintenance of the arachnoid barrier.

Moreover, if the LMCs undergo stress in culture, it is crucial to establish better culture methods that ensure the cells are in a similar state to their *in vivo* counterparts. This will help in performing more complex experiments on LMCs and better understand their role as brain barrier cells.

Future work should focus on further characterizing the LMCs to develop a more representative barrier of *in vivo* conditions. This will enable the researchers to perform more complex experiments on the LMCs and gain a better understanding of their role as a brain barrier cell.

## 4 Conclusion

In summary, the brain barriers play a critical role in maintaining the proper environment in the brain and protecting it from harmful substances. Dysfunction of the cells within these barriers can lead to various neurological disorders, underscoring their importance in CNS health. However, the barriers formed by the meninges that regulate CSF interaction with the brain and blood vessels in the dura mater and subarachnoid space have received relatively little attention compared to the BBB and BCSFB. Recent studies have just begun to reveal the significance of these meningeal barriers, from their role in regulating the immune system's response to brain injuries to their involvement in the glymphatic system's waste removal from the CSF. These advances have highlighted the crucial interactions between the CSF, meningeal barriers, and the brain in both health and disease, emphasizing the need for further research in this field.

The present study aimed to investigate the cell type of the primary LMC and the characteristics of the LMCs in culture in terms of morphology, proteomics, and barrier formation. Specifically, we examined whether the cells were derived from the pia or arachnoid, whether cell culture and culture techniques had an effect on LMCs and their responses. The results of our study indicate that the cells could be a co-culture of pial and arachnoid LMCs, necessitating further analysis for experimental use. Furthermore, we found that cell passage is a variable that can significantly alter the morphology and protein expression of LMCs as early as passage five. Our findings also show a loss of epithelial cell properties and apparent loss of barrier characteristics, as evidenced by low TEER values and no junctional E-cadherin or occludin localisation. Similar observations have been reported in other studies that performed TEER on LMCs.

## 5 Methods

**Fig. 11:**
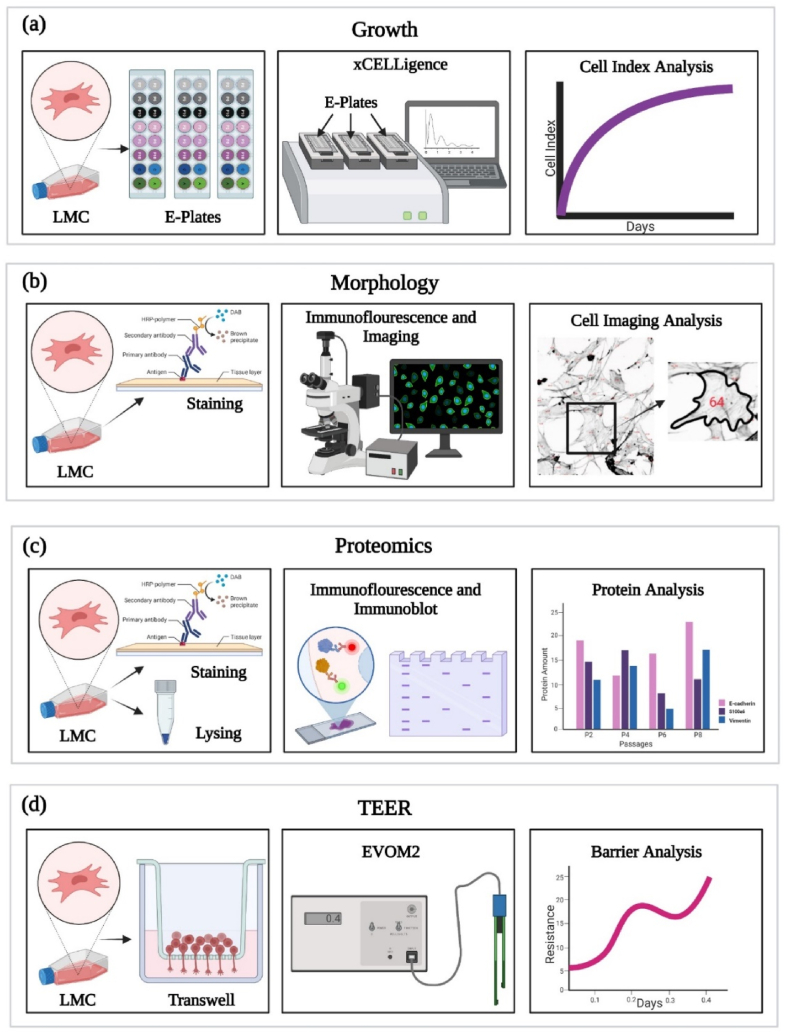
(A) Leptomeningeal cell growth analysis was completed using real-time cell analysis. (B) Morphology of the leptomeningeal cells were characterized through DAPI and Phalloidin staining to assess cell shape at the different passages. (C) Leptomeningeal cell proteomics was assessed through immunoblotting to compare specific protein expression at the different passages. (D) Leptomeningeal cell barrier formation was measured using TEER to compare the overall barrier formation over 16 days and the difference depending on passage number. (Colour). (For interpretation of the references to colour in this figure legend, the reader is referred to the Web version of this article.)

An overview of the methods used in this study are illustrated in Fig. 11. These methods, described in detail below, were utilized to assess the difference in growth, morphology, protein expression and barrier strength in LMCs at different passages.

### 5.1 Leptomeningeal cell culture

A vial of primary human LMC were obtained [#1400, Sciencell] and cultured per supplier recommendation. This consisted of growing the cells in Poly-L-Lysine (PLL) coated T75's at a concentration of 2 μg/cm^2^ in complete growth meningeal medium [#1401, Sciencell] containing 5% meningeal growth serum, 2% fetal bovine serum (FBS) and 5% Penicillin-Streptomycin (Pen-Strep). The cells were sub-cultured by washing the cells in Dulbecco's phosphate-buffered saline (DPBS) [D8537, Sigma-Aldrich] twice and dissociating the cells in 1:1 DPBS and 0.25% Trypsin-EDTS [T4049, Sigma-Adrich] dilution. Cells were then centrifuged at 1000 RPM for 5 min. The supernatant was then discarded, and cell count completed using a LUNA™ counter [L10001-LG, Logos] at a 1:1 Trypan blue to cell concentration dilution. A density of 5000 cell/cm^2^ was reseeded and the remaining cell frozen down in 50% meningeal medium, 40% FBS and 10% dimethyl Sulfoxide (DMSO). Vials for each passage were stored in liquid nitrogen until all passages were collected.

### 5.2 Leptomeningeal cell growth analysis

A real-time cell analysis (RTCA) system was used to measure LMCs cell growth [xCELLigence, Agilent Technologies Inc] (Fig. 11a). The RTCA system evaluates cell growth based on cell impedance and outputs a Cell Index (CI) value which is influenced by cell attachment, spreading, and/or proliferation [40]. Background measurement was taken by placing 100 μL of medium into the electronic plates and acquiring a CI reading. Three experimental replicates and 6 technical replicates were set up to assess LMC cell growth at different concentrations for passage 2. The LMCs were seeded at cell concentrations of 1.5x10^4^ cell/mL, 3x10^4^ cell/mL and 9x10^4^ cell/mL on either non-coated wells, wells that were coated in 2ug/cm^2^ Poly-l-lysine (PLL) [Thermofisher] and wells coated in 5ug/cm^2^ Collagen Rat-tail [Corning] to compare the effect of coating on cell growth. A similar set-up was used for determining growth at different LMC cell passages. For this test, the manufacturers recommended concentration of 3x10^4^ cell/mL was used to evaluate growth as passage two, passage five and passage ten on either non-coated wells, wells that were coated in of PLL and Collagen. Again, wells containing media were used as a blank control. For data analysis, the B–CI (Baseline Cell Index) is determined by subtracting the CI for a cell-containing well from the CI of the blank control.

### 5.3 Immunofluorescence

LMCs were stained for Vimentin [MAB2105, Biotechne], pan-Cytokeratin [AB7753, Abcam], Desmoplakin I + II [sc-390975, Santa-Cruz], CRABP2 [NB19051-7h12, Biotechne], S100A6 [NB100-2590, Biotechne], E-cadherin [SAB4503751, Sigma], Connexin-43 [AB11370, Abcam] and Occludin [SAB3500301, Sigma]. LMCs were seeded at a density of 5000 cells/cm^2^ on glass cover slips and left to grow until full confluency was reached. The cells were fixed in 4% paraformaldehyde (PFA) in 1x phosphate buffer solution (PBS) for 15 min at room temperature, followed by permeabilization in buffer made up of 0.1% Triton x-100 for the intercellular markers and 0.1% Tween-20 for the extracellular-junctional markers in 1x PBS for 5 min at room temperature. This was followed by incubation in blocking buffer made up of 5% FBS in 1x PBS for 1 h at room temperature. Primary antibodies were diluted in the blocking buffer and left overnight 4 °C. The secondary antibody diluted in the blocking buffer were applied on the next day and left for 1 h at room temperature in the dark. Phalloidin [**Invitrogen]** and DAPI [Invitrogen] were applied following 3 washes with 1x PBS and incubated for 20 min and 10 min respectively. Coverslips were mounted onto glass slides using mounting agent [Prolong Diamond Antifade Mountant, Fisher Scientific Ltd] and imaged using an IMX confocal high-content imaging system [Molecular Devices] at 20x and 40x widefield magnification (Fig. 11b). Exposure was constant for all images that are compared (Supplementary Table S3).

Twenty-five images were acquired for each experimental condition and analysed using a image processing software specific for cell analysis (Cell Profiler 4.2.1). Phalloidin of each LMC for each passage was used to assess the cell morphology based on eccentricity, form factor, area and solidity [92](Supplementary Figure S12). The image processor identifies the cell based on nuclei position and extracts the area and shape feature of each cell [93]. The mean of the twenty five images for each experimental condition was compared. Similarly, the intensity of the specific proteins (Vimentin, CRABP2, S100a6, and E-Cadherin) was assessed using the measure object intensity module. The average pixel intensity of each cell within an image was found and normalized by dividing by the image maximum possible intensity value. The mean intensity value for the twenty five images were compared.

### 5.4 Immunoblotting

Immunoblotting was carried out similar to the method described in Ross et al. [94]. For immunoblotting experiment, LMCs were collected and lysed on ice for 30 min. Protein content was quantified using a bicinchonic acid assay (BCA) [Fisher Scientific].

Samples containing 15 μg of protein were prepared with 1x LDS sample buffer [Invitrogen] and 2.5% β-mercaptoethanol [Fisher Scientific] and heated to 95 °C for 5 min. Samples were run on 4–12% Bis-Tris gels [Invitrogen] using 1x MES running buffer [Invitrogen]. Gels were transferred to nitrocellulose membranes [GE Healthcare] in 1x MES transfer buffer [Invitrogen]. Vimentin and Cytokeratin gels were run for 1 h at 100V in 4 °C. Membranes were blocked for 1 h at room temperature in 5% skim milk in tris-buffer saline-0.1% tween 20 and probed overnight at 4 °C with primary antibody (Supplementary Table S1). The membranes were incubated for 1 h at room temperature with secondary antibody (Supplementary Table S2). Membranes were imaged using the UVITEC Q9 Alliance (Fig. 11c). Triplicate experiments were conducted. Densitometry was conducted using FIJI image J [FIJI 2.9.0]. The intensity of the proteins were normalized against GAPDH. The mean normalized relative intensity was calculated across the triplicate experiments (Supplementary Figure S10-S11).

### 5.5 Barrier formation analysis

The transendothelial/Transepithelial electrical resistance (TEER) was completing following a similar approach to Ross et al. [94]. The apical side of a 0.4 μm pore, polycarbonate 12-well transwell [Corning 3401] was seeded with the manufacturer recommended cell density. LMC medium was changed every 3 days. The TEER of the cell barrier were measured every day using the EVOM2 [World-Precision Instruments] as described by Yeste et al. [95]. The resistance of a blank well (membrane with no cells) was recorded as a background reading and subtracted from the measured resistance values. This value was multiplied by the membrane surface area (1.12 cm^2^) to calculate the TEER reading. Three measurements were acquired for each well per day for sixteen days. Passages were evaluated in triplicate with three plates in total (Fig. 11d). TEER results are presented as the mean ± standard deviation. Brightfield images of the LMCs at full confluency are provided in the supplementary file (Figure S13).

### 5.6 Statistical analyses

All statistical analysis was carried out using Prism 6 software Version 9 (GraphPad, U.S.A.). All data sets were tested for normality using the Shapiro-Wilk test. The values for each experiment were expressed as the mean ± SEM. For normally distributed data, values were compared using two-way ANOVA with a 95% confidence interval and Bonferroni multiple comparison test. For non-parametric data sets, values were using the Kruskal-Wallis test at a 95% confidence interval with Dunn's multiple comparison test. All error bars indicate standard deviation of the mean. P < 0.05 was considered significant.

## Data availability

The datasets used and/or analysed during the current study are available from the corresponding author on reasonable request.

## Funding

Irish Research Council Funding GOIPG/2020/1424.

## CRediT authorship contribution statement

**Mannthalah Abubaker:** Writing – review & editing, Writing – original draft, Visualization, Methodology, Investigation, Formal analysis, Data curation, Conceptualization. **Aisling Greaney:** Software, Methodology, Investigation. **David Newport:** Writing – review & editing, Supervision, Resources, Project administration, Conceptualization. **John J.E. Mulvihill:** Writing – review & editing, Supervision, Resources, Project administration, Conceptualization.

## Declaration of competing interest

The authors declare that they have no known competing financial interests or personal relationships that could have appeared to influence the work reported in this paper.

## List of Abbreviations

BBB: Blood brain barrier
BCSFB: Blood Cerebrospinal fluid barrier
BCA: Bicinchoninic acid assay
CI: Cell Index
CSF: Cerebrospinal Fluid
DMSO: Dimethylsulfioxide
DPBS: Dulbecco's phosphate buffered saline
EMT: Epithelial-mesenchymal transition
LMCs: Leptomeningeal cells
MECs: Meningothelial cells
PLL: Poly-L-Lysine
PFA: Paraformaldehyde
RTCA: Real time cell analysis
TEER: Transendothelial/Transepithelial electrical resistance
