# Supplementary material for: Characterization of primary human leptomeningeal cells in 2D culture
Source: Heliyon. 2024 Feb 20;10(5):e26744. doi: 10.1016/j.heliyon.2024.e26744 (PMC10906397; doi:10.1016/j.heliyon.2024.e26744)
Supplement: Multimedia component 1 [file mmc1.docx]

**Supplementary Material**

1. **Primary Antibodies**

**Table S.1:** Summary of the Primary antibodies used for immunoblotting and immunofluorescence.

| **Primary** | **Product Code** | **Dilution IB** | **Dilution IF** |
| --- | --- | --- | --- |
| Vimentin | Biotechne: 280618MAB2105 | 3/1000 | 3/250 |
| Pan-Cytokeratin | Sigma: SAB5300264 | 1/1000 | 1/500 |
| Desmoplakin I+II | Santa-Cruz: sc-390975 | - | 1/200 |
| S100A6 | Biotechne: nbp1-89388 | - | 1/200 |
| CRABP2 | Biotechne: nb100-2590 | - | 1/500 |
| E-cadherin | Sigma: SAB4503751 | - | 1/200 |
| Connexin-43 | Merck: C6219 | - | 1/400 |
| Occludin | Sigma: SAB3500301 | - | 1/200 |
| GAPDH | Sigma: SAB530025 | 1/5000 | - |

1. **Secondary Antibodies**

**Table S.2:** Summary of the Secondary antibodies used for immunoblotting and immunofluorescence.

| **Secondary Immunoblot** | **Product Code** | **Dilution** |
| --- | --- | --- |
| Anti-Rat in Goat | Abcam: ab175778 | 1/10,000 |
| Anti-Mouse in Goat | Invitrogen: A11375 | 1/10,000 |
| Anti-Rabbit in Goat | Invitrogen: A11375 | 1/10,000 |
| **Secondary Immunofluorescence** | **Product Code** | **Dilution** |
| Anti-Rat in Goat | Invitrogen: A48262 | 1/1000 |
| Anti-Mouse in Goat | Abcam: Ab150115 | 1/1000 |
| Anti-Rabbit in Goat | Abcam: Ab175471 | 1/1000 |

1. **Exposure**

**Table S.3:** Summary of exposures used for each protein for mean intensity analysis.

| **Protein** | **Magnification** | **Exposure** |
| --- | --- | --- |
| Vimentin | 20x | 116.57 |
| Pan-Cytokeratin | 20x | 285.87 |
| S100A6 | 20x | 499.57 |
| CRABP2 | 20x | 499.57 |
| E-cadherin | 20x | 2070.88 |
| Connexin-43 | 20x | 207088 |

1. **XCELLigence Raw Data:**

**
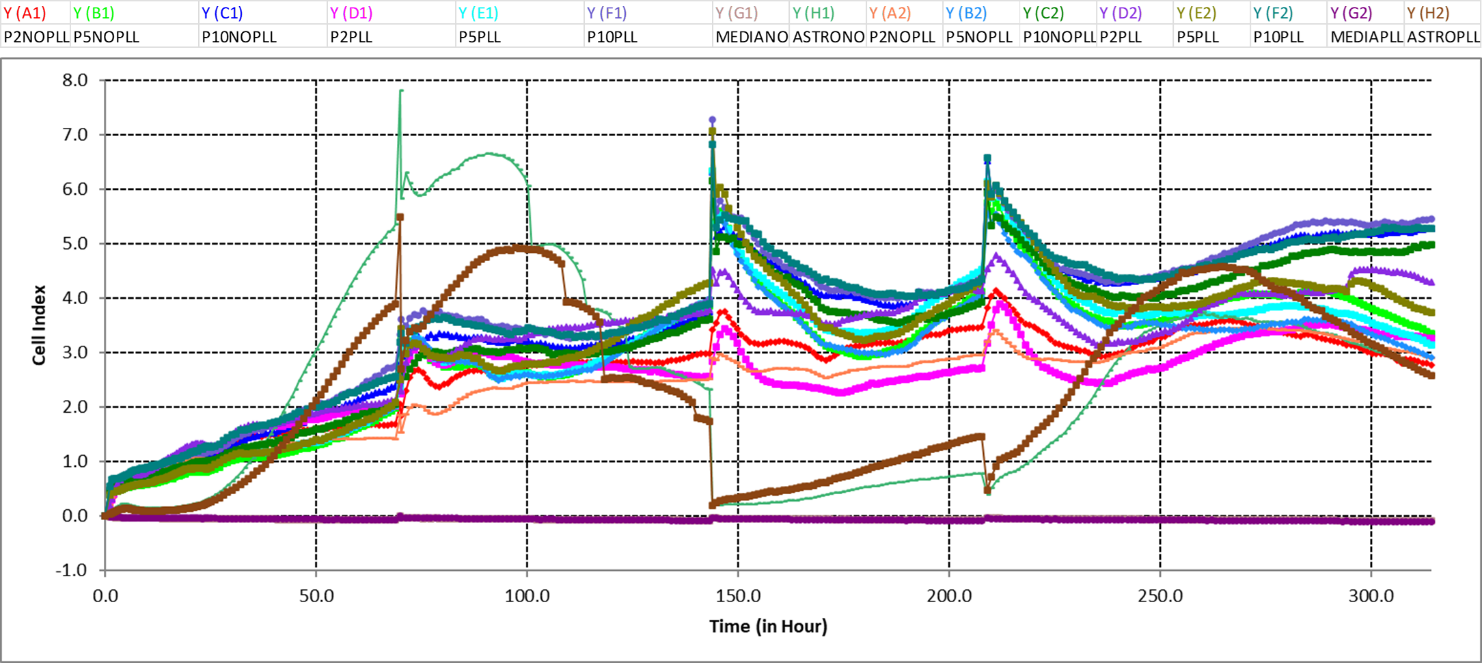
**

**Figure S. 1:** Raw data from the xCELLigence for e-Plate 1/3 for passage 2, passage 5 and passage 10 seeded on PLL and Plastic


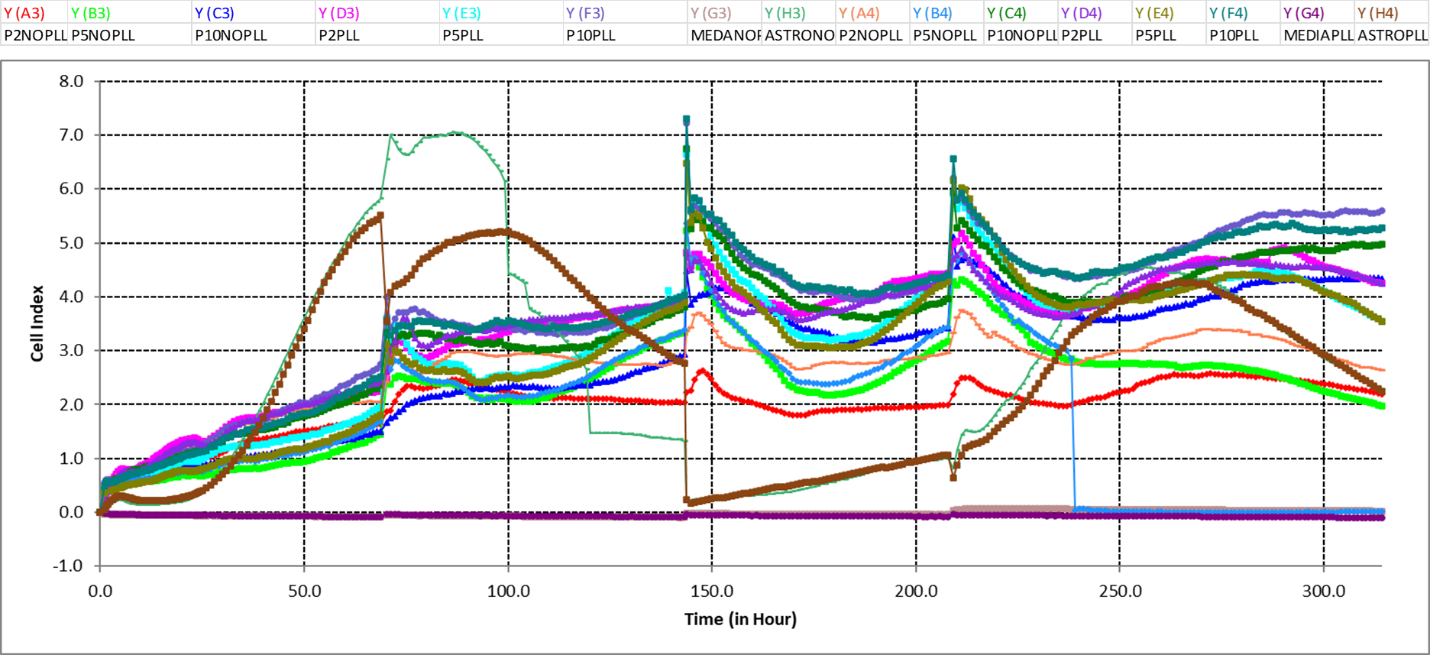


**Figure S. 2:** Raw data from the xCELLigence for e-Plate 2/3 for passage 2, passage 5 and passage 10 seeded on PLL and Plastic
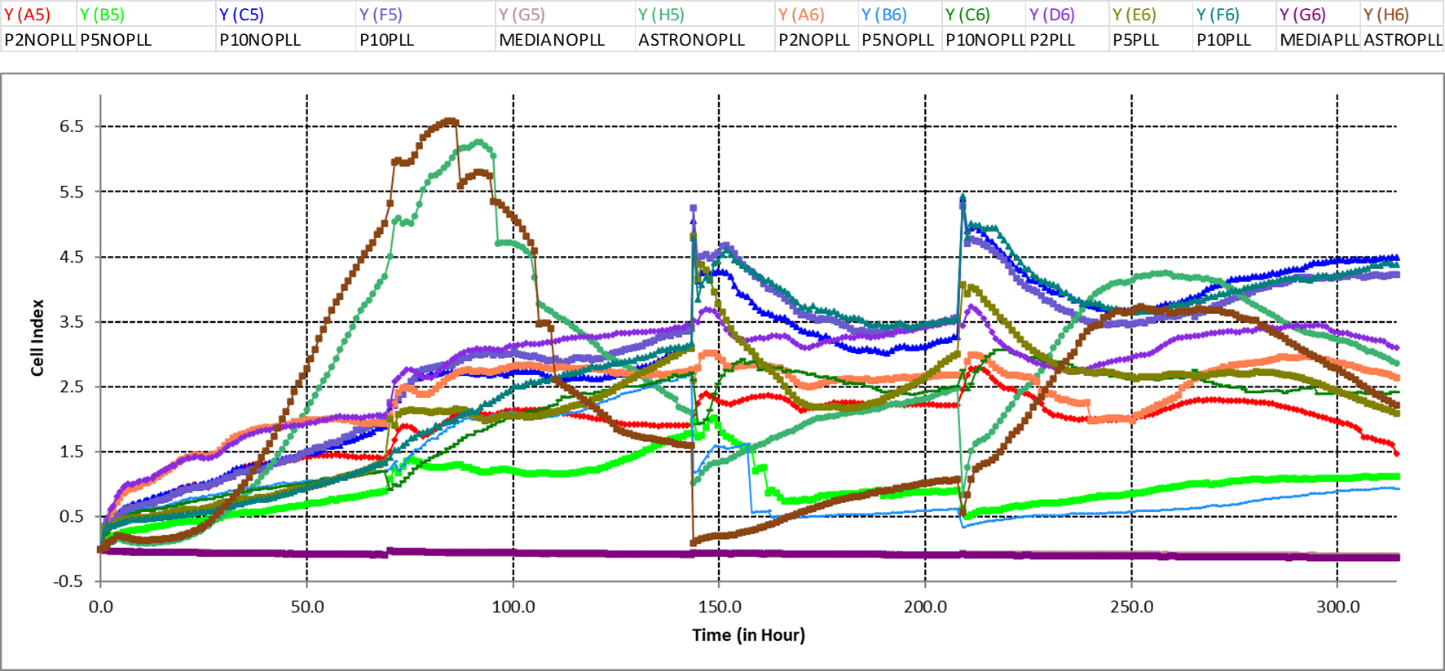


**Figure S. 3:** Raw data from the xCELLigence for e-Plate 3/3 for passage 2, passage 5 and passage 10 seeded on PLL and Plastic


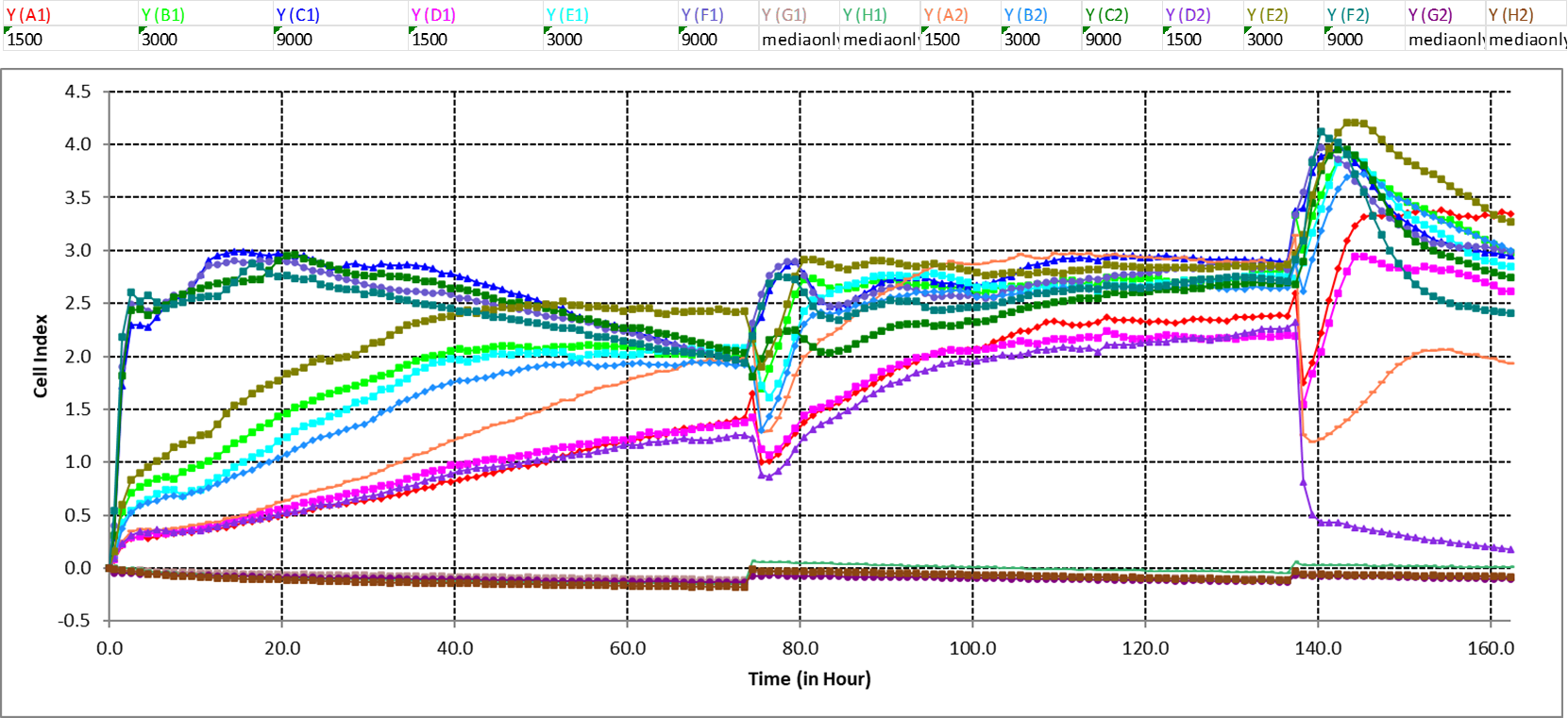


**Figure S. 4:** Raw data from the xCELLigence for e-Plate 1/3 for examining the effect of concentrations 1.5x10^4^ cell/mL , 3x10^4^ cell/mL and 9x10^4^ cell/mL seeded on PLL and Plastic


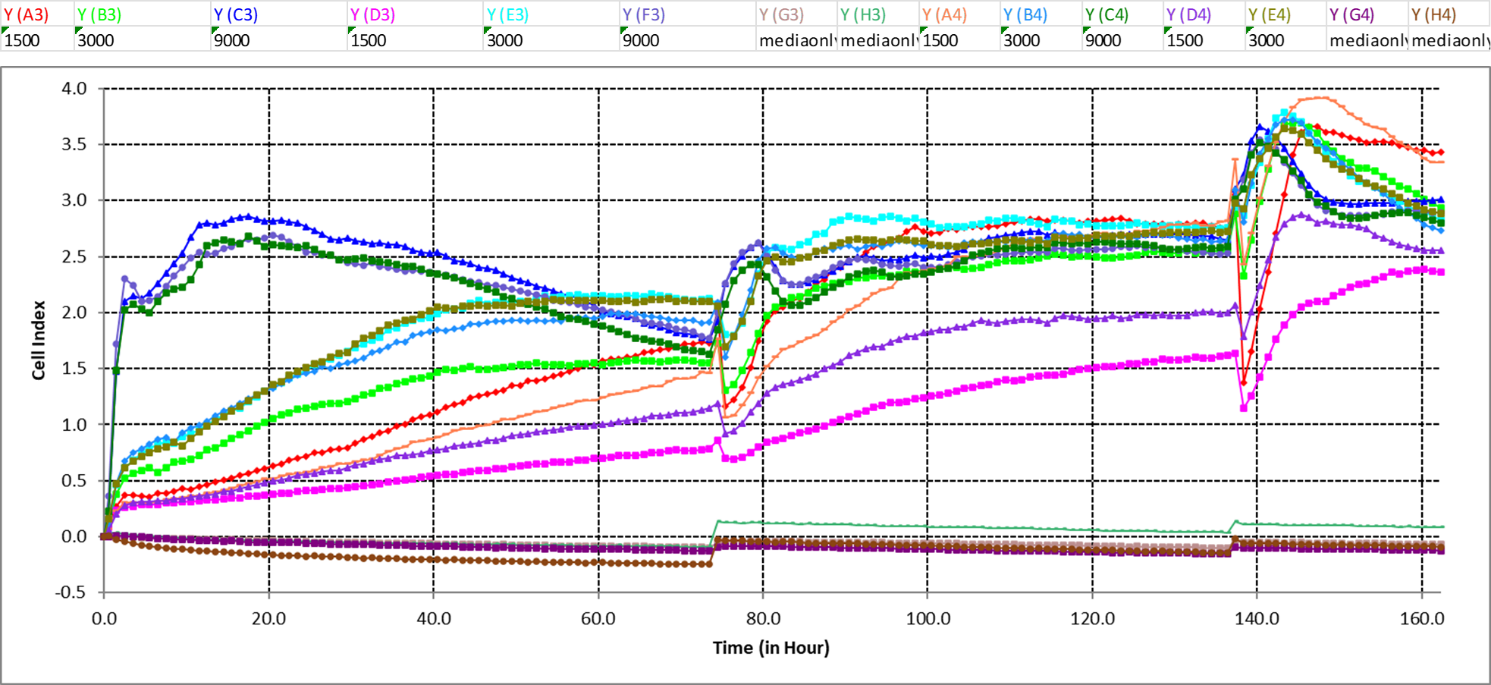


**Figure S. 5:** Raw data from the xCELLigence for e-Plate 2/3 for examining the effect of concentrations 1.5x104 cell/mL , 3x104 cell/mL and 9x104 cell/mL seeded on PLL and Plastic


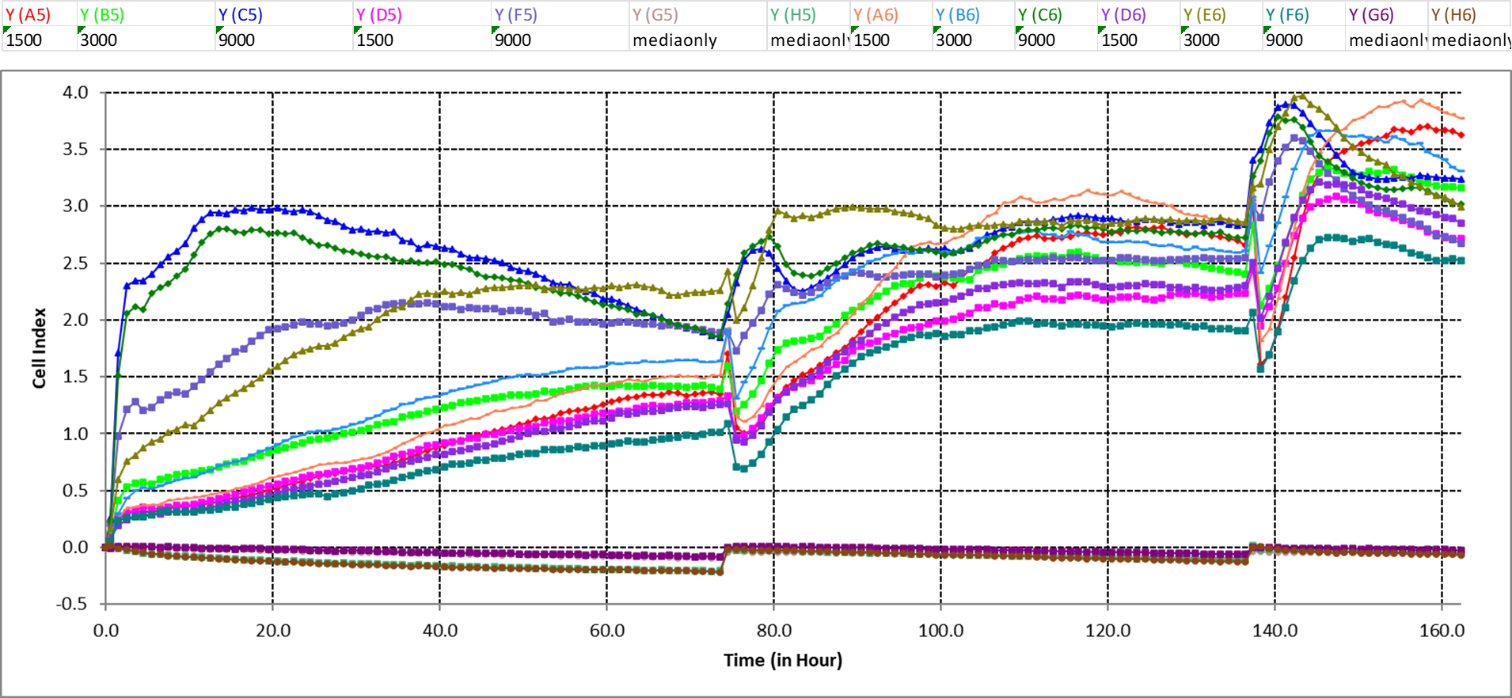


**Figure S. 6:** Raw data from the xCELLigence for e-Plate 3/3 for examining the effect of concentrations 1.5x10^4^ cell/mL , 3x10^4^ cell/mL and 9x10^4^ cell/mL seeded on PLL and Plastic


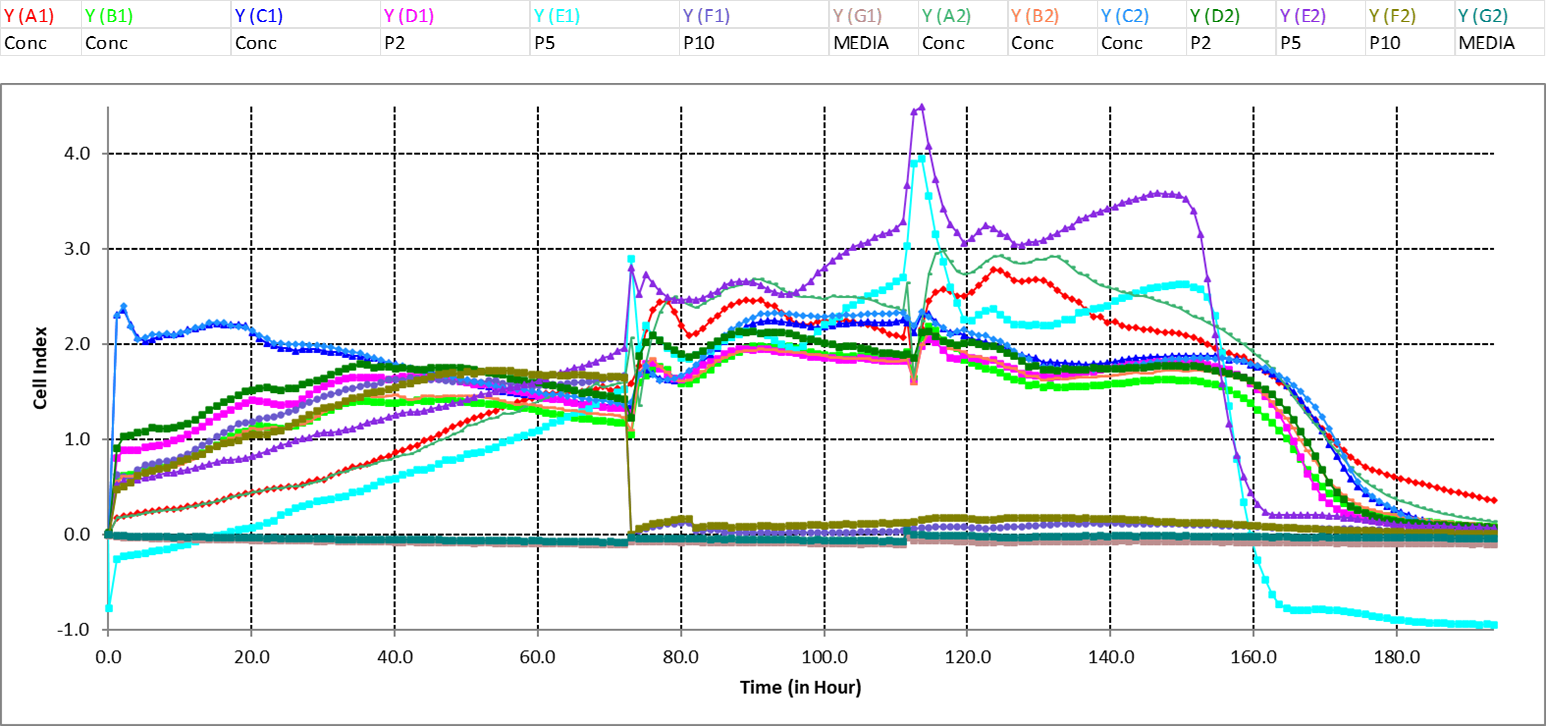


**Figure S. 7:** Raw data from the xCELLigence for e-Plate 1/3 for passage 2, passage 5 and passage 10 and for examining the effect of concentrations 1.5x10^4^ cell/mL , 3x10^4^ cell/mL and 9x10^4^ cell/mL seeded on collagen.

**
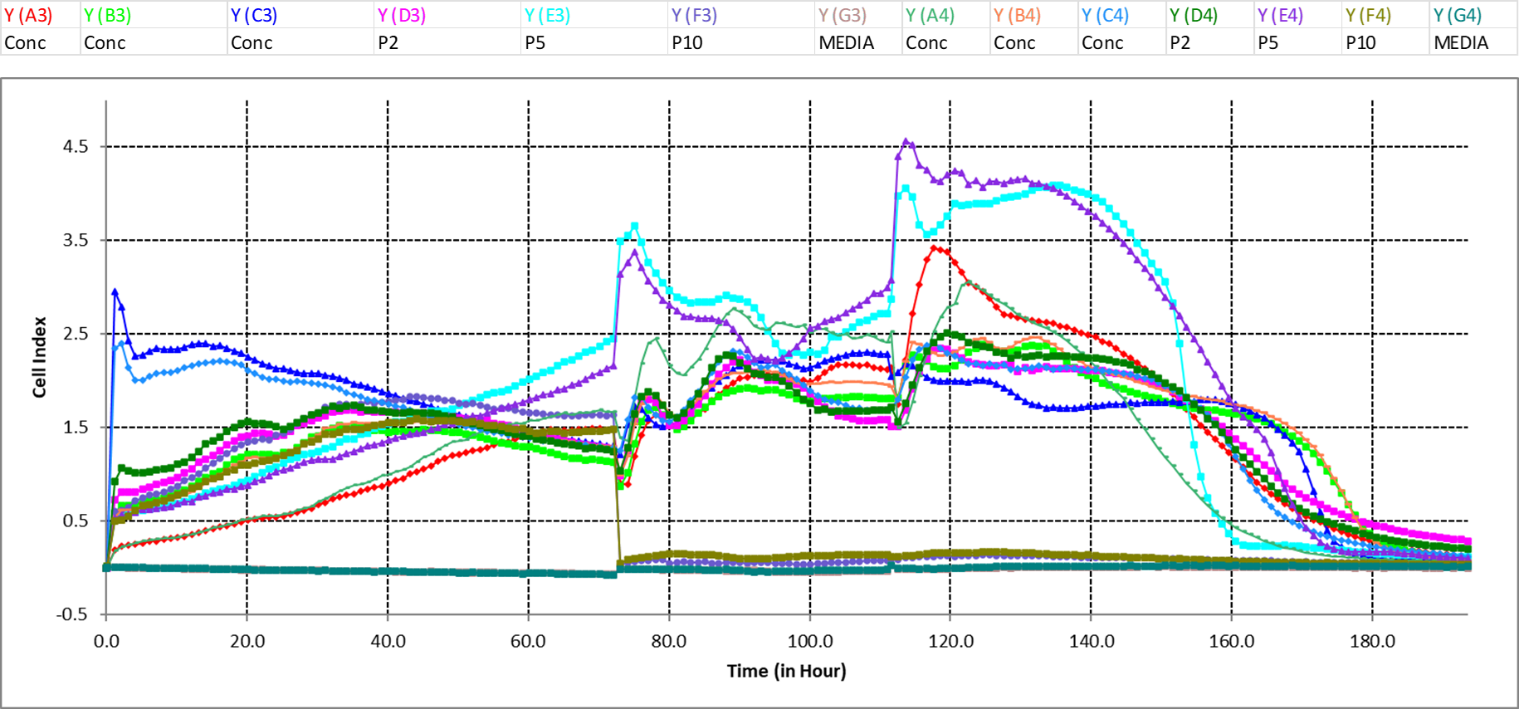
**

**Figure S. 8:** Raw data from the xCELLigence for e-Plate 2/3 for passage 2, passage 5 and passage 10 and for examining the effect of concentrations 1.5x10^4^ cell/mL , 3x10^4^ cell/mL and 9x10^4^ cell/mL seeded on collagen.


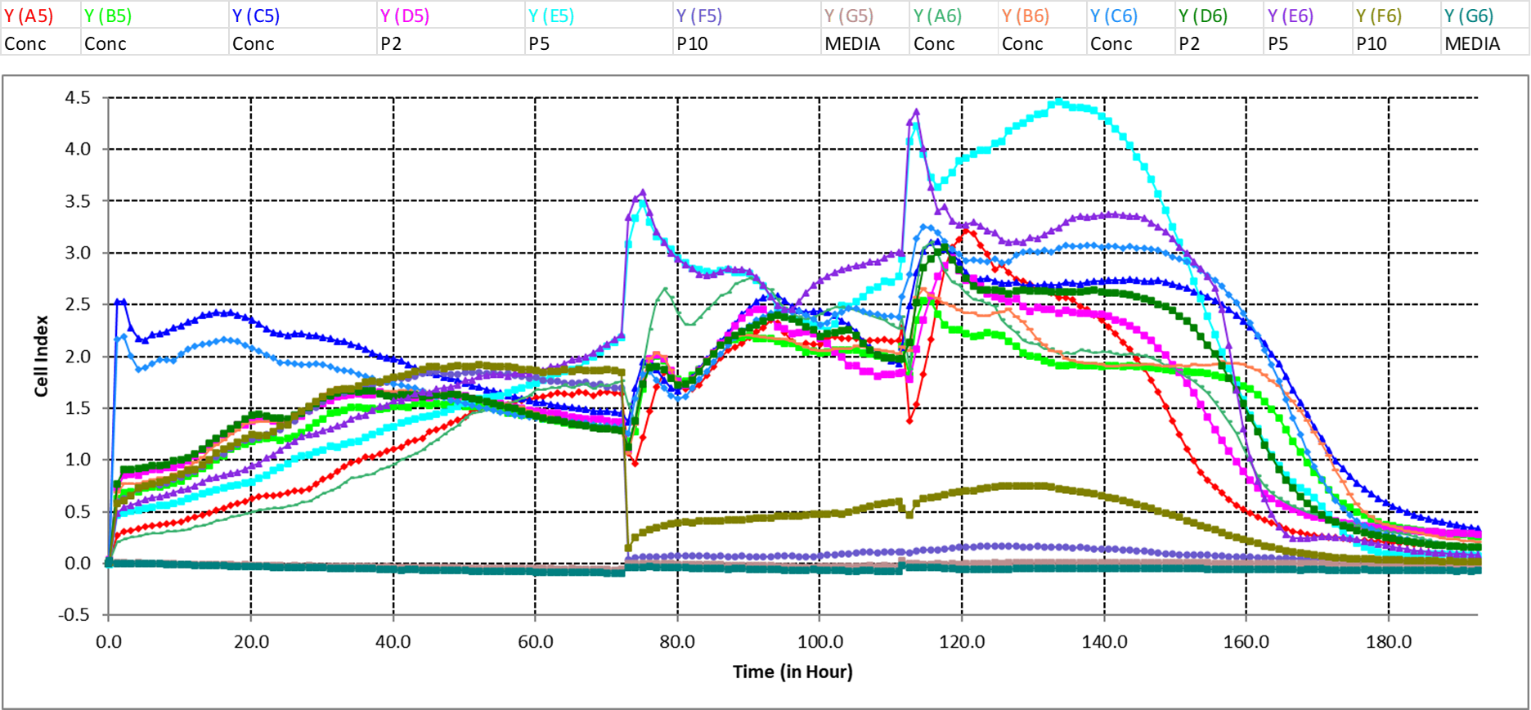


**Figure S. 9:** Raw data from the xCELLigence for e-Plate 3/3 for passage 2, passage 5 and passage 10 and for examining the effect of concentrations 1.5x10^4^ cell/mL , 3x10^4^ cell/mL and 9x10^4^ cell/mL seeded on collagen.

1. **Western blots:**

**
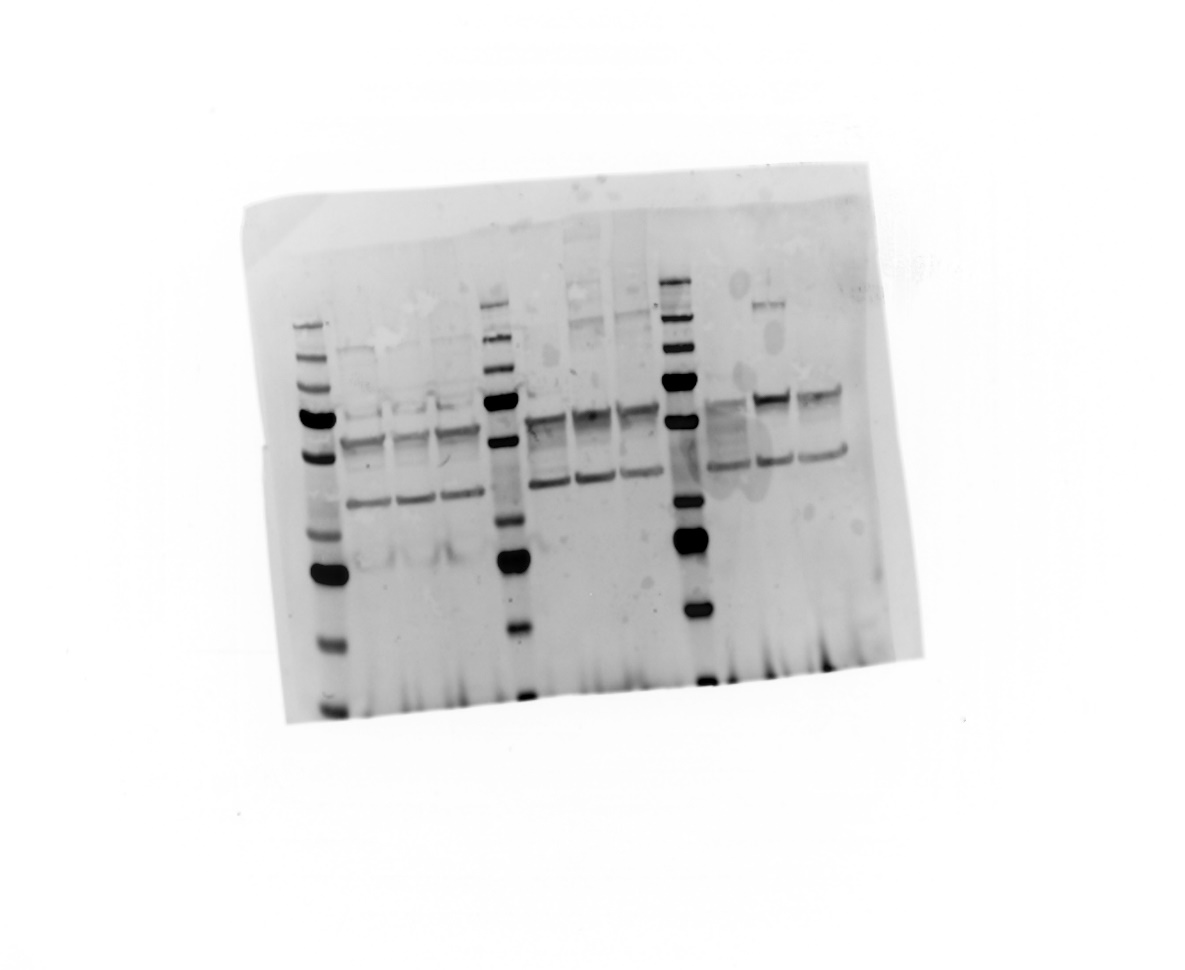
**

**Figure S. 10:** Vimentin 55 kDAa and GAPDH 35 kDa for P2, P5 and P10 repeated in triplicates.

**
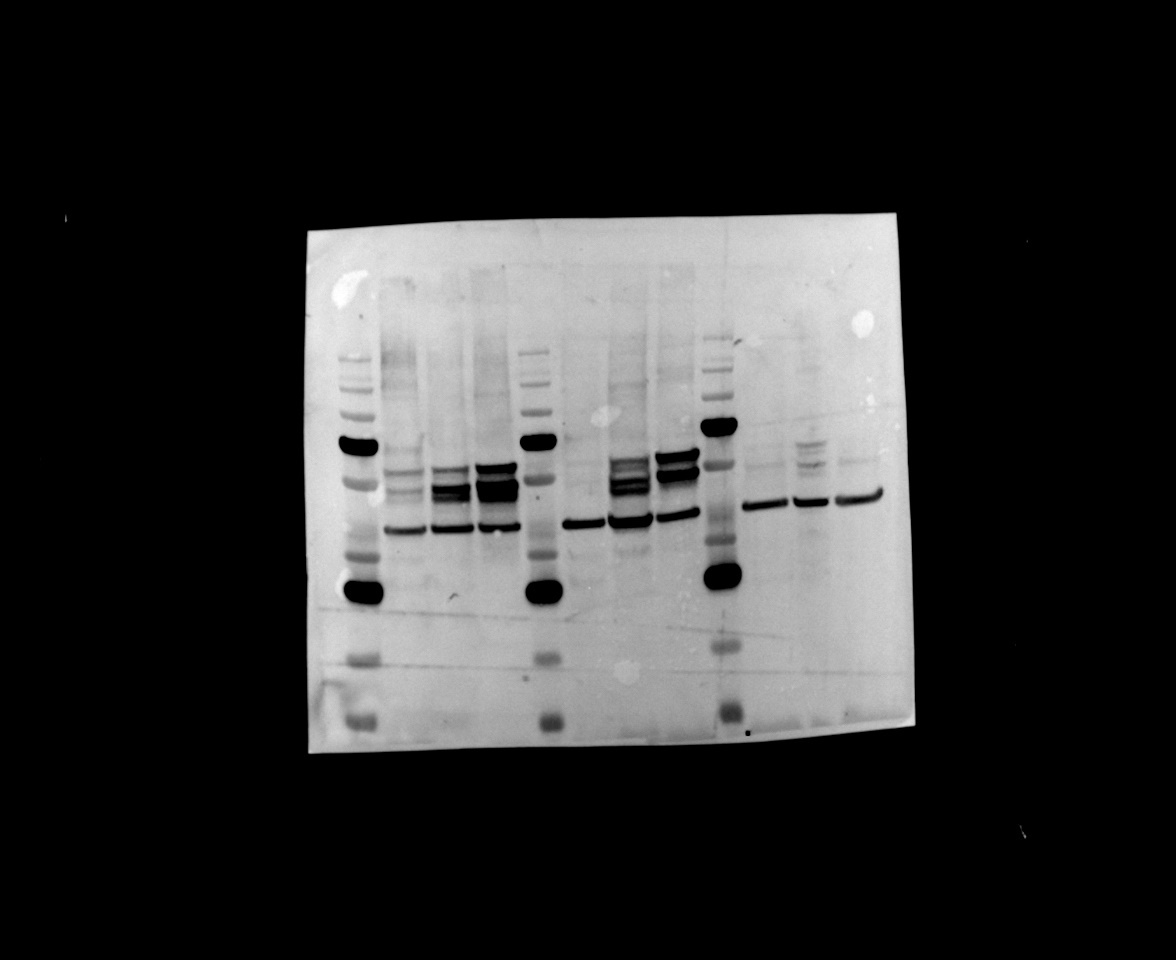
**
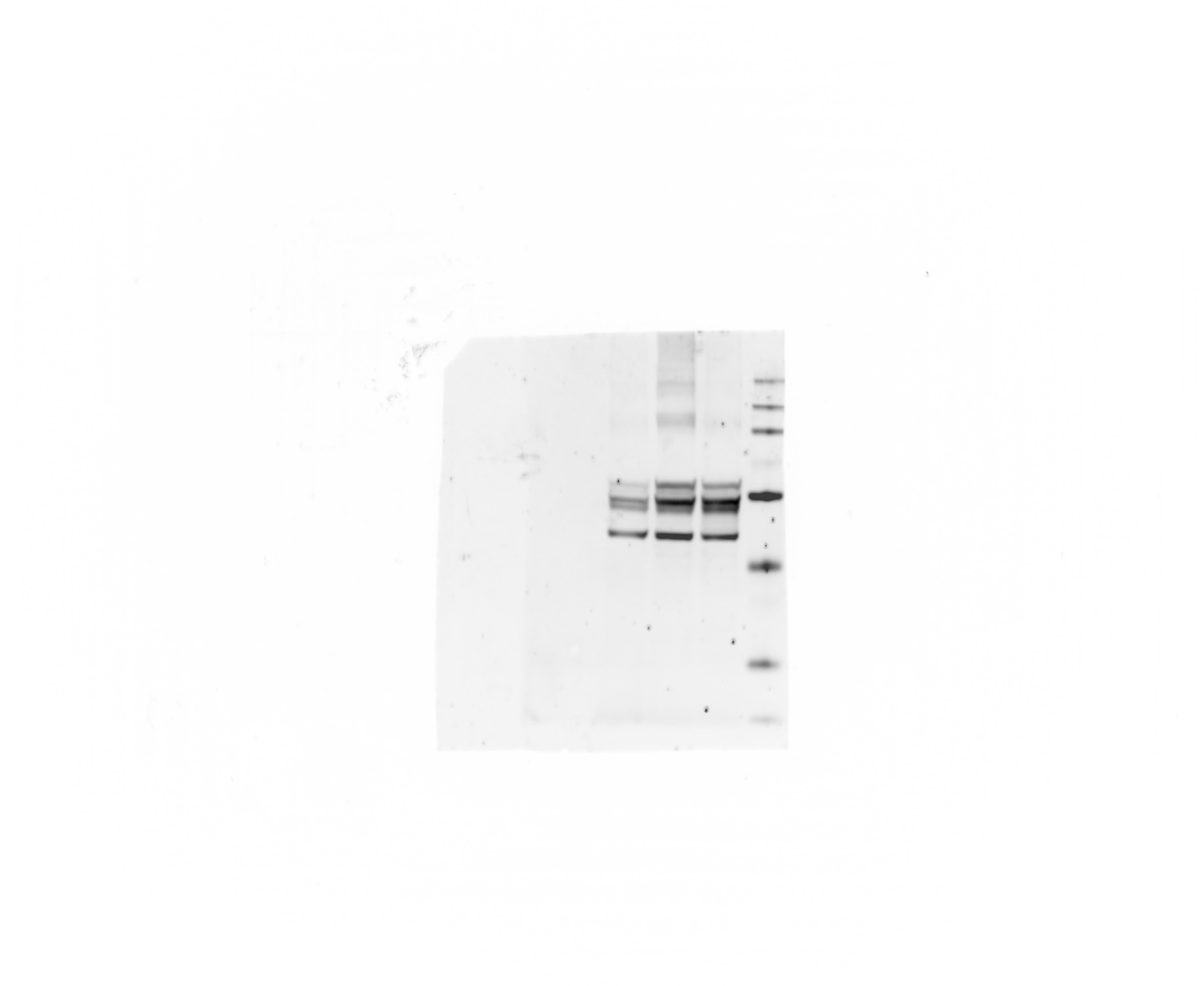


**Figure S. 11:** pan-Cytokeran and GAPDH 35 kDa for P2, P5 and P10 repeated in triplicates.

1. **Immunofluorescence:**
   1. **Image Acquisition:**

All images were acquired using automated confocal imaging on the Molecular Devices ImageXpress

High-Content Microconfocal. Focusing and exposure optimization were carried out on a small subset

of wells across the range of the plate. The sites used to optimize the imaging conditions were not

acquired for image analysis to avoid photobleaching which could affect the images used for

quantitation. Automated imaging uses rapid, plate bottom- and well bottom-based autofocusing to

ensure each individual site is in focus before acquiring the images. Any potential bleaching caused by

autofocusing would be negligible as the DAPI channel is used and it is uniformly applied to all wells

and sites. All images were acquired at the same magnification, exposure, and laser intensity.

- 1. **Image Analysis using CellProfiler**


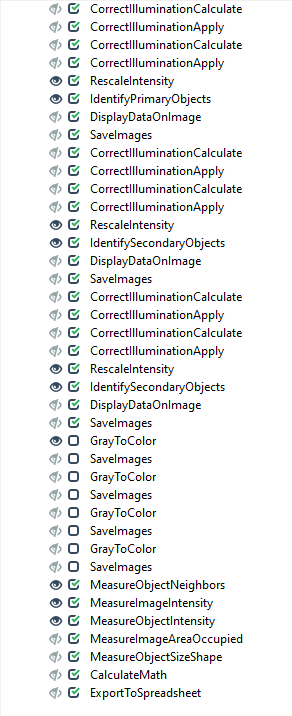


**Figure S. 12:** Cell profiler Pipeline used to obtain mean intensity values for the proteins of interest

**
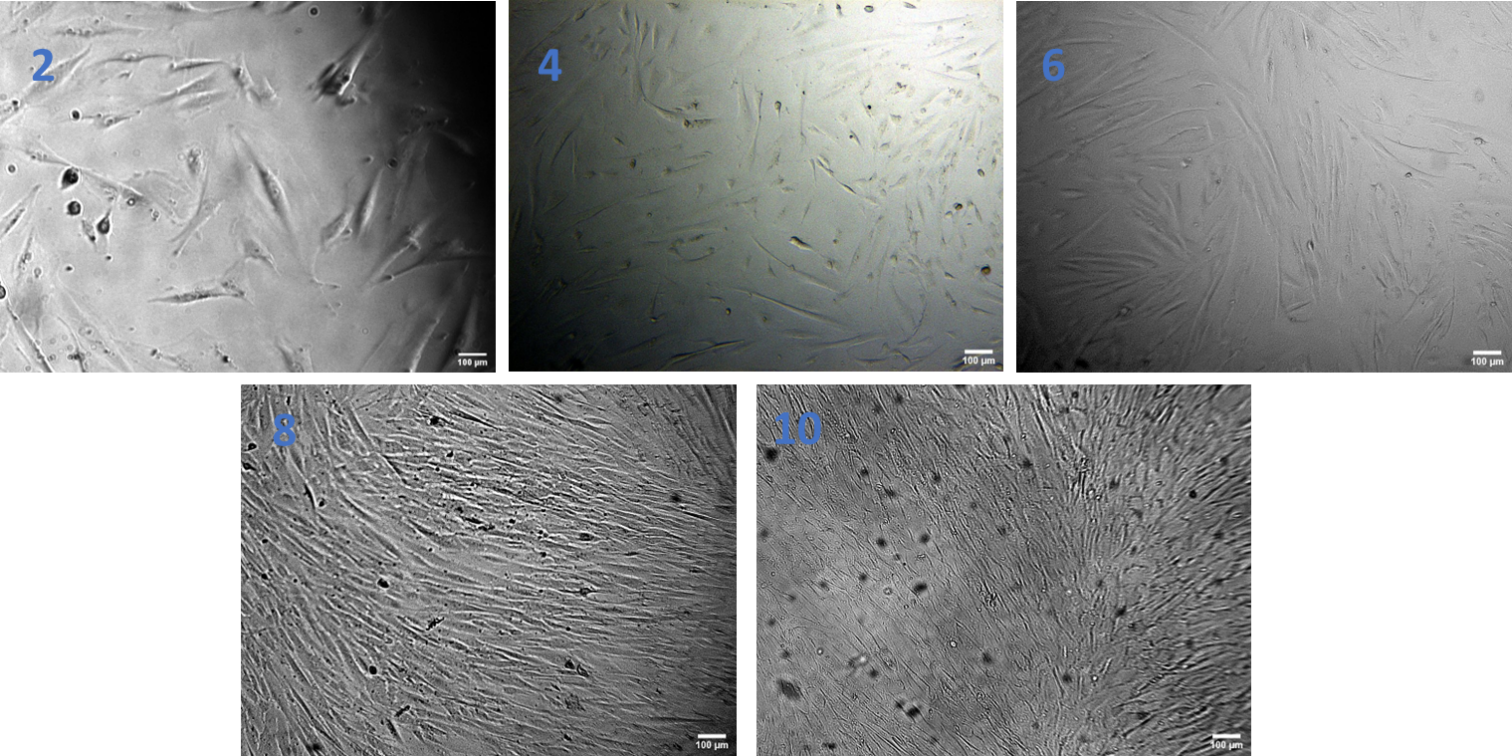
**

**Figure S. 13:** Brightfield images at 4x magnification over 10 days. LMCs reach full confluency at day 6 to 7


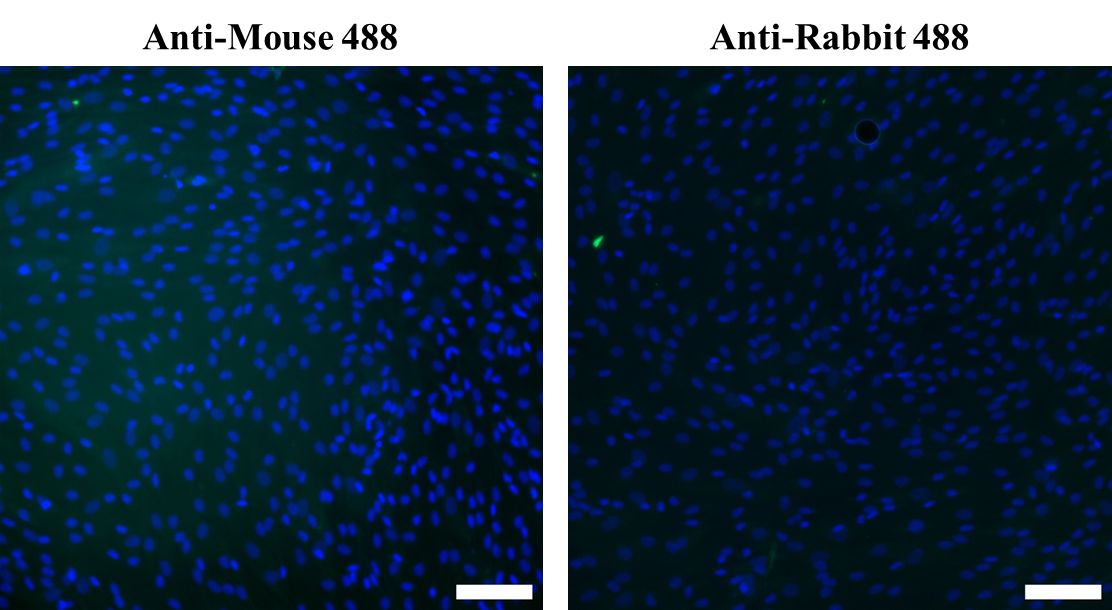


Figure S. 14: Immunofluorescence images of LMCS stained with secondary only antibodies as negative control.
